# Thermoregulatory ability and mechanism do not differ consistently between neotropical and temperate butterflies

**DOI:** 10.1111/gcb.16797

**Published:** 2023-06-14

**Authors:** Benita C. Laird‐Hopkins, Esme Ashe‐Jepson, Yves Basset, Stephany Arizala Cobo, Lucy Eberhardt, Inga Freiberga, Josh Hellon, Gwen E. Hitchcock, Irena Kleckova, Daniel Linke, Greg P. A. Lamarre, Alex McFarlane, Amanda F. Savage, Edgar C. Turner, Ana Cecilia Zamora, Katerina Sam, Andrew J. Bladon

**Affiliations:** ^1^ Institute of Entomology Biology Centre of the Czech Academy of Sciences České Budějovice Czech Republic; ^2^ Faculty of Science University of South Bohemia České Budějovice Czech Republic; ^3^ Smithsonian Tropical Research Institute Panama City Panama; ^4^ Department of Zoology University of Cambridge Cambridge UK; ^5^ Maestria de Entomologia University of Panama Panama City Panama; ^6^ Department of Biosciences Durham University Durham UK; ^7^ Wildlife Trust of Bedfordshire, Cambridgeshire, and Northamptonshire Cambourne UK

**Keywords:** behaviour, climate change, ecophysiology, ectotherms, insects, Lepidoptera, microclimate, tropics

## Abstract

Climate change is a major threat to species worldwide, yet it remains uncertain whether tropical or temperate species are more vulnerable to changing temperatures. To further our understanding of this, we used a standardised field protocol to (1) study the buffering ability (ability to regulate body temperature relative to surrounding air temperature) of neotropical (Panama) and temperate (the United Kingdom, Czech Republic and Austria) butterflies at the assemblage and family level, (2) determine if any differences in buffering ability were driven by morphological characteristics and (3) used ecologically relevant temperature measurements to investigate how butterflies use microclimates and behaviour to thermoregulate. We hypothesised that temperate butterflies would be better at buffering than neotropical butterflies as temperate species naturally experience a wider range of temperatures than their tropical counterparts. Contrary to our hypothesis, at the assemblage level, neotropical species (especially Nymphalidae) were better at buffering than temperate species, driven primarily by neotropical individuals cooling themselves more at higher air temperatures. Morphology was the main driver of differences in buffering ability between neotropical and temperate species as opposed to the thermal environment butterflies experienced. Temperate butterflies used postural thermoregulation to raise their body temperature more than neotropical butterflies, probably as an adaptation to temperate climates, but the selection of microclimates did not differ between regions. Our findings demonstrate that butterfly species have unique thermoregulatory strategies driven by behaviour and morphology, and that neotropical species are not likely to be more inherently vulnerable to warming than temperate species.

## Graphical abstract

We studied how temperate and neotropical butterflies respond to temperature change. Neotropical butterflies were better at regulating their body temperature than temperate butterflies, mainly because at high air temperatures neotropical butterflies cooled themselves more. However, the ability to do this was determined by a butterfly's wing size and shape (generally longer and narrower in neotropical species), rather than its thermal environment. Temperate butterflies raised their body temperature more than tropical butterflies, probably because they live in cooler climates. Butterflies have different ways of regulating their temperature and neotropical butterflies are not necessarily more vulnerable to climate change than temperate butterflies.

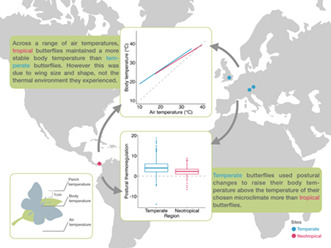

## Abstracta

El calentamiento global es una gran amenaza para las especies alrededor del mundo, sin embargo, no se tiene bien definido sí en los insectos, las especies distribuídas en las zonas tropicales son más vulnerables a los cambios de temperature que las especies de zonas templadas o viceversa. Para responder a este interrogante, utilizamos un protocolo de campo estandarizado aplicado a especies de mariposas distribuídas en zonas tropicales (Panamá) versus zonas templadas (Reino Unido, República Checa y Austria), con el cual buscamos: (1) Evaluar la capacidad de amortiguación (capacidad de regular la temperatura corporal en relación con la temperatura del aire circundante) en el a nivel de ensamblaje y familia, (2) Determinar sí las diferencias en la capacidad de amortiguación es facilitada por sus características morfológicas, y (3) Investigar cómo las mariposas usan los microclimas y el comportamiento para termorregularse a tráves de mediciones de temperatura ecológicamente relevantes. Nuestra hipotesis incial soportaba que las mariposas templadas estaban adaptadas para amortiguar los cambios de temperatura en comparación con las mariposas neotropicales, ya que las especies templadas experimentan un rango más amplio de temperaturas que sus contrapartes tropicales. Contrariamente a nuestra hipótesis, a nivel de ensamble, las especies neotropicales (especialmente familia Nymphalidae) fueron mejores en la capaicidad de amortiguacion que las especies templadas, explicado por el hecho de que individuos se enfrían más a altas temperaturas del aire. Así, la morfología fué el principal impulsor de las diferencias en la capacidad de amortiguación entre las especies neotropicales y templadas en comparación con el ambiente térmico experimentado por las mismas. Encontramos que las mariposas templadas utilizaron la termorregulación de postura para elevar su temperatura corporal más que las mariposas neotropicales, probablemente como una adaptación a los climas templados, aunque la selección de microclimas no difirió entre regiones. Nuestros hallazgos demuestran que las especies de mariposas tienen estrategias de termorregulación únicas, impulsadas principalmente por el comportamiento y morfología, además nuestros resultados demuestran que a diferencia de lo que se ha pensado, las especies neotropicales son igual de vulnerables al calentamiento de su hábitat que las especies templadas.

## 1 INTRODUCTION

Climate change is predicted to cause a rise in mean global temperatures, with larger increases at higher latitudes, as well as increases in the frequency of extreme temperature events (Lee et al., 2021). Temperature changes can cause shifts in physiology, morphology, life history and distribution of species (Angilletta, 2009; Franco et al., 2006; Parmesan, 2006; Sunday et al., 2014). Considering this, the effect of temperature change on species is and will be wide‐reaching, with potential cascading effects to communities and ecosystems (Eggleton, 2020; Parmesan, 2006; Sunday et al., 2014). Consequently, there is an urgent need to understand how species respond to changing temperatures.

To understand and predict the impacts of climate change on communities, it is important to identify the traits that affect species' sensitivity to temperature (Diamond et al., 2011; Diamond & Yilmaz, 2018). These include morphological traits, such as size or colouration, as well as physiological and behavioural traits, and may be a consequence of the thermal environment a species inhabits (Bonebrake et al., 2014; González‐Tokman et al., 2020; Stella et al., 2018; Wenda et al., 2021). On a global scale, a major difference between thermal environments is found between temperate and tropical regions, with a narrower temperature range and higher mean annual temperature in tropical regions than temperate regions (Lee et al., 2021). Janzen (1967) predicted that the lower temperature variation found in tropical compared to temperate regions may select for narrower thermal tolerances among tropical species (the ‘seasonality’ hypothesis). Thus, although the degree of warming is predicted to be greater at higher latitudes, tropical species may be more at risk to temperature changes than temperate species, as they are less able to cope with variability (Lee et al., 2021). In addition, tropical species live closer to their thermal maximum than temperate species, and therefore may be more vulnerable to the same level of warming (Deutsch et al., 2008; Huey et al., 2009; Sunday et al., 2019). As a result, small variations in temperature would have severe consequences for the survival and fitness of tropical species (Deutsch et al., 2008; Ghalambor, 2006). However, other studies have shown that some temperate species may be equally vulnerable to climate change (Colado et al., 2022; Johansson et al., 2020; Mi et al., 2022). For example, temperate species with short activity periods may only experience narrow ranges in temperature (Johansson et al., 2020) and may be selected for low thermal tolerance in a similar way to tropical species (Colado et al., 2022). Despite more than 50% of insects species being found in the tropics, there is a lack of systematic field studies investigating the thermoregulatory capabilities of tropical ectotherms, and comparing tropical and temperate species (Deutsch et al., 2008; Johansson et al., 2020; Stork, 2018; Sunday et al., 2012). It remains unclear the extent to which species from different ecological backgrounds and latitudes differ in their sensitivity to climate change.

The impact of climate change on species is often tested at the macroclimate scale, using coarse‐scale weather station data, assuming that species live at these ambient temperatures (Diamond & Yilmaz, 2018; Pincebourde & Woods, 2020). This does not take into consideration the small‐scale microclimate differences in temperature that individual species experience in heterogenous environments, nor the fact that for small ectotherms moving just a small distance in these environments can result in exposure to very different climatic conditions (Pincebourde & Woods, 2020). Indeed, temperatures between microclimates and weather stations can differ significantly, with weather stations overestimating annual mean temperature in tropical understory forest by up to 0.5°C and in highlands by up to 2.0°C (Montejo‐Kovacevich et al., 2020). Small‐scale temperature variation can play an important role in species' thermal adaption, and can both buffer or magnify the effects of increasing temperatures (Pincebourde & Woods, 2020). We lack knowledge of how rising temperatures at the macroclimate scale impact microclimate temperatures, and how species are affected by these fine scale differences in temperature.

Butterflies are important as an indicator taxa, pollinators, prey and herbivores, but as ectotherms may be particularly vulnerable to temperature change (Bonebrake et al., 2010; Diamond & Yilmaz, 2018; Ma et al., 2021; Menéndez et al., 2007). Understanding how they respond to temperature change is crucial for assessing the ecosystem‐wide impacts of climate change (Harvey et al., 2020). Butterflies can regulate their body temperature in a variety of ways, including by selecting favourable thermal microclimates during different life stages or at different times of day (Cómbita et al., 2022; De Frenne et al., 2021; Kearney et al., 2009; Kleckova et al., 2014). They also use other behavioural mechanisms, such as the orientation of their wings relative to the sun, to increase or reduce thermal absorbance (Kemp & Krockenberger, 2002; Shanks et al., 2015). Morphological characteristics, including wing colour, reflectance, size and hair length, can also be important in determining thermoregulatory ability (Bonebrake et al., 2014; Stella et al., 2018; Wenda et al., 2021). For example, light‐coloured butterflies can regulate their body temperature by using their wings to reflect solar radiation onto their thorax to heat up, or back into the environment to cool down (Shanks et al., 2015; Zeuss et al., 2014). Dark‐coloured butterflies can heat up quicker than light‐coloured butterflies, perhaps explaining why they are found more frequently in cooler climates, such as forest interiors or higher latitudes, where being able to heat up quickly increases fitness (Günter et al., 2019; Xing et al., 2016). Size can also determine thermoregulatory ability, with the thermoregulation of sympatric mountain butterflies differing between species in relation to their habitat use and body size (Kleckova et al., 2014). Large species can use the surface area of their wings to intercept sunlight and are generally better at thermoregulating than small species (Bladon et al., 2020; Wenda et al., 2021). Finally, thermoregulatory ability differs systematically between butterfly families, with temperate Pieridae species being particularly good at thermoregulating compared to other temperate families (Bladon et al., 2020).

In this study, we compared buffering ability—the ability to regulate body temperature independently of the surrounding air temperature—of butterfly populations from neotropical (Panama) and temperate regions (Czech Republic, Austria and the United Kingdom). We hypothesised (H1) that butterflies from temperate regions, being naturally exposed to a greater range of temperatures, will be better able to buffer their body temperature against changes in air temperate than those from the neotropical region. We tested this at the assemblage and family level. Since there was significant overlap in butterfly species found at our European sites, and at our Panamanian sites, we treated them as single assemblages, resulting in a temperate group and a neotropical group of the most common and conspicuous butterflies in each region. If differences in buffering ability between neotropical and temperate regions were observed we hypothesised (H2) that this will be driven by the temperatures butterflies are exposed to in the different regions, not by differences in their morphological traits. Finally, we hypothesised (H3) that as an adaptation to temperate climates, temperate butterflies will actively select warmer microclimates than neotropical butterflies, and that they will heat up through postural means more than neotropical species.

## 2 METHODS

### 2.1 Study sites

Neotropical data were collected in Panama from February to June 2020 and from October 2021 to March 2022 during both wet (May–December) and dry (January–April) seasons (Figure S1; Table S1) (Leigh, 1999). Temperate data were collected in the Czech Republic and Austria between April and August 2021 and in the United Kingdom between April and September 2009 and May and September 2018 (Figure S1; Table S1) (Bladon et al., 2020). Data collection took place between 7:30 and 17:30. Neotropical field sites included lowland scrub and managed urban green spaces, secondary semi‐deciduous lowland tropical forest, mountain rainforest and management agroforestry (Table S1). Temperate field sites included calcareous meadows, grassland meadows, alpine/montane grassland, encroaching scrub, secondary forest and exposed ground (Table S1).

### 2.2 Butterfly body temperature and morphological measurements

Butterflies were captured with butterfly nets when encountered (without chasing) and data were collected following the protocol used by Bladon et al. (2020), as follows. Once in the net, and within 10 s, a temperature reading of the butterfly's thorax (body temperature, *T*_b_) was taken using a thermocouple (0.5 mm diameter) and handheld indicator (Tecpel Thermometer 305B, TC Direct). Air temperature (*T*_a_) was taken at waist height where the butterfly was caught, with the thermocouple shaded from the sun. If the butterfly was resting on a substrate before capture, the temperature of the air 1 cm above where it was sat was recorded with the thermocouple (microclimate temperature, *T*_m_). The butterfly was identified to species or subspecies. In the case of butterflies from the tropical *Calephelis* genus it was not possible to identify individuals to species, so data from these butterflies were aggregated to genus level. Forewing length (in mm) from the tip of the wing to the point where it meets the thorax was measured using callipers (at the Panama and UK sites only).

We calculated mean forewing length from our field data for each of the Panama and UK species. With the exception of *Erebia* spp., for which there was field data (Laird‐Hopkins, unpublished data), mean forewing lengths of butterflies from the Czech Republic and Austria were taken from the literature (Lindsey, 2016).

Wing aspect ratio (the ratio of forewing length: forewing width) strongly influences flight performance, which in turn can determine a species' capacity to move to more favourable microclimates and hence their ability to buffer air temperature (Chazot et al., 2016). Wing aspect ratio of all neotropical and temperate species was calculated from photographs sourced from the literature (Table S2). To do this, photographs of five female and five male mounted specimens of each species were sourced and uploaded into ImageJ (Schneider et al., 2012). The forewing length (as above) and depth (longest line between the leading and trailing edges of the forewing, measured perpendicular to the wing length axis) were measured, and used to calculate forewing aspect ratio for each specimen. Mean aspect ratio was then calculated for each species. For sexually dimorphic species, the mean forewing aspect ratio of females and males was calculated separately.

Colour scale was determined for each species based on the lightness/darkness of their wings, following Bladon et al. (2020) (ranked: 1—white, 2—yellow‐green, 3—orange, 4—orange‐brown/blue, 5—brown and 6—black). If the difference in colour between males and females was sufficient to classify them in different categories, the mean colour category of females and males was used.

### 2.3 Statistical analysis

#### 2.3.1 Preliminary analysis

To ensure our estimates of buffering ability were robust, only species with at least 10 *T*_b_ measurements across a range of at least 5°C of *T*_a_ were included in analyses. To estimate the buffering ability of each species, a simple linear regression with *T*_b_ as the response variable and *T*_a_ as the predictor variable was fitted separately for each species. The slope of the relationship was extracted and, following Bladon et al. (2020), subtracted from one to provide an estimate of buffering ability (buttering ability estimate). With this method, a steep slope shows that the butterfly's *T*_b_ varies greatly over small ranges of *T*_a,_ thus indicating a low buffering ability. A shallow slope shows that the butterfly's *T*_b_ remains relatively stable over wide ranges of *T*_a,_ thus indicating a high buffering ability.

#### 2.3.2 Do temperate and neotropical butterfly assemblages and families differ in buffering ability?

In total 94 species, across six families (Hesperiidae, Lycaenidae, Nymphalidae, Papilionidae, Pieridae and Riodinidae) were sampled in our study regions (neotropical: 54 species, *n* = 1333; temperate: 40 species, *n* = 5482). There is only one Riodinidae species (*Hamearis lucina*) found in Europe, so our analysis for this family specifically compared this species' buffering ability to those of its neotropical relatives. Papilionidae species were only sampled in the neotropical region, so this family was removed when comparing the buffering ability within families across neotropical and temperate regions.

To test for differences in buffering ability between butterfly assemblages, and within each butterfly family, in neotropical and temperate regions, linear mixed effects models were fitted for the assemblage and for each family separately. Body temperature was fitted as the response variable and *T*_a_, region (temperate or neotropical) and their two‐way interaction as predictor variables. Species was included as a random effect. A likelihood ratio test was performed to determine if the model with the two‐way interaction was a better fit than the model without the term; if so, it would suggest that butterfly assemblages, or families, in the two regions differ in their buffering ability. The slope estimates for each region (neotropical and temperate) were extracted and subtracted from one as described above, to obtain a mean buffering ability estimate across each assemblage and family in each region.

#### 2.3.3 When controlling for morphology, do temperate and neotropical butterflies differ in buffering ability?

Morphological characteristics are known to be important in determining species' thermoregulatory abilities (Bladon et al., 2020; Shanks et al., 2015; Sheldon & Tewksbury, 2014; Xing et al., 2016). We first determined if morphological characteristics (mean forewing length, mean forewing aspect ratio and wing colour) differed between neotropical and temperate species. We fitted one‐way ANOVAs with mean forewing length (mm), mean wing aspect ratio and wing colour separately as the response variable and region (neotropical or temperate) as the predictor variable.

To determine if forewing length, forewing aspect ratio and/or wing colour were driving differences in buffering ability between neotropical and temperate butterflies we fitted linear mixed effects models. Body temperature (*T*_b_) was the response variable. Air temperature (*T*_a_), region (temperate or neotropical), mean forewing length, mean forewing aspect ratio and wing colour, and the two‐way interactions between *T*_a_ and each of the other terms were predictor variables. Mean forewing length was log_10_‐transformed to ensure all predictor variables were on a similar scale. Species was included as a random effect. If the interaction between *T*_a_ and region was retained in the best‐fitting model, it would suggest that buffering ability differs between regional assemblages in addition to any effect of morphology. To find the best‐fitting model we used backward stepwise selection with the step function in R package ‘*lmerTest*’ (Kuznetsova et al., 2017). A preliminary Pearson correlation coefficient analysis indicated no correlation between the three continuous predictor variables (mean forewing length, mean forewing aspect ratio and wing colour), justifying their inclusion as predictor variables in the same models (Panel S1). Preliminary analysis fitting one‐way ANOVAs with mean forewing length (mm), mean wing aspect ratio and wing colour separately as the response variable and family as the predictor variable showed that there was a significant effect of family on the three response variables (Table S3). Family was therefore not included in the linear mixed effects model described above.

#### 2.3.4 Do temperate and neotropical butterfly assemblages and families differ in their use of microclimate selection and postural thermoregulation, or in their index of thermal specialisation?

The data were subset so that only species with at least 10 microclimate temperature measurements (*T*_m_) were included in this analysis. For the assemblage‐level analysis, this included 39 species, 13 neotropical (*n* = 176; families: five Hesperiidae, seven Nymphalidae and one Riodinidae) and 26 temperate (*n* = 919; families: three Hesperiidae, four Lycaenidae, 15 Nymphalidae and four Pieridae) (Table S4). For the family‐level analysis there were only sufficient data from both temperate and neotropical regions for Hesperiidae and Nymphalidae to undertake this analysis. This included five neotropical and three temperate Hesperiidae (neotropical: *n* = 57; temperate: *n* = 56), and seven neotropical and 15 temperate Nymphalidae (neotropical: *n* = 101; temperate: *n* = 499).

For each individual butterfly, we calculated the extent to which its chosen *T*_m_ differed from the surrounding *T*_a_, by subtracting *T*_a_ from *T*_m_ (‘microclimate selection’, following Bladon et al. (2020)). A large difference between *T*_m_ and *T*_a_ indicates that the butterfly is selecting a specific thermal microclimate distinct from *T*_a_. For each butterfly, we also calculated the extent to which *T*_b_ differed from the temperature of its chosen *T*_m_, by subtracting *T*_m_ from *T*_b_ (‘postural thermoregulation’, following Bladon et al. (2020)). A large difference between *T*_m_ and *T*_b_ suggests that the butterfly is regulating its temperature independently of its chosen microclimate, for example by behavioural posturing relative to the sun. To quantify the relative contribution of microclimate selection and postural thermoregulation to each butterfly's body temperature, we calculated an index of thermal specialisation (ITS) by subtracting microclimate selection (*T*_m_–*T*_a_) from postural thermoregulation (*T*_b_–*T*_m_). A large ITS value indicates that the butterfly is utilising postural thermoregulation more, and is able to alter its body temperature beyond that of its immediate environment, making it a thermal generalist. A small ITS value means that the butterfly relies more on microclimate selection for thermoregulation, and is a thermal specialist (Bladon et al., 2020).

To test whether the use of microclimate selection and postural thermoregulation, and the value of ITS, differed between the butterfly assemblage and within butterfly families from neotropical and temperate regions, linear mixed effects models were fitted for the assemblage, and each family separately. Microclimate selection, postural thermoregulation and ITS were fitted separately as response variables, and region (temperate or neotropical), *T*_a_ and their two‐way interaction were included as predictor variables. Species was included as a random effect. A likelihood ratio test was performed to determine if the full model was a better fit for the data than a model without the interaction term between region and *T*_a_; if so, it would suggest that butterflies from different regions differed in their use of microclimate selection, postural thermoregulation or ITS across a range of *T*_a_. An additional likelihood ratio rest was performed to determine if the model including region and *T*_a_ as a predictor variable was a better fit than the model including only *T*_a_; if so, it would suggest that butterflies from different regions differed in their use of microclimate selection, postural thermoregulation or ITS, but independently of *T*_a_.

To control for differences in *T*_a_ between neotropical and temperate regions and to assess the robustness of our results, all analyses (1, 2 and 3) were repeated on a subset of data that were restricted to the same *T*_a_ range in both neotropical and temperate regions. All statistical analyses were undertaken in R version 4.1.2 (R Core Team, 2019). Graphics were produced using the package ‘ggplot2’ (Wickham, 2016). Linear mixed effects models were fitted using the ‘lme4’ package (Bates et al., 2015). To ensure normality and homogeneity in the residuals, graphical model diagnostics were applied to each model. The response parameter was log_10_‐transformed where necessary to achieve normality. Marginal means for neotropical and temperate species and families were predicted using the package ‘ggeffects’ (Lüdecke, 2018). The marginal effects of region, that is, the difference between marginal means, were used as a measure of effect size at the response scale.

## 3 RESULTS

From the neotropical region, 1333 individuals from 54 species were measured. Air temperature (*T*_a_) ranged from 17.4°C to 39.7°C, with a mean of 28.5°C. Body temperature (*T*_b_) ranged from 22.1°C to 40.3°C, with a mean of 31.7°C. From the temperate region, 5482 individuals of 40 species were measured (Results S1). *T*_a_ ranged from 10.0°C to 34.8°C, with a mean of 22.2°C. *T*_b_ ranged from 15.4°C to 45.0°C, with a mean of 28.0°C. The buffering ability estimates for neotropical species ranged from −0.317 (*Hemiargus hanno*) to 1.005 (*Phoebis argante*), with a mean of 0.295 (Figure S2; Tables S4 and S5). The buffering ability estimates for temperate species ranged from −0.404 (*Hamearis lucina*) to 0.675 (*Erebia medusa*), with a mean of 0.243 (Figure S2; Table S4).

### 3.1 Do temperate and neotropical butterfly assemblages differ in buffering ability?

**FIGURE 1:**
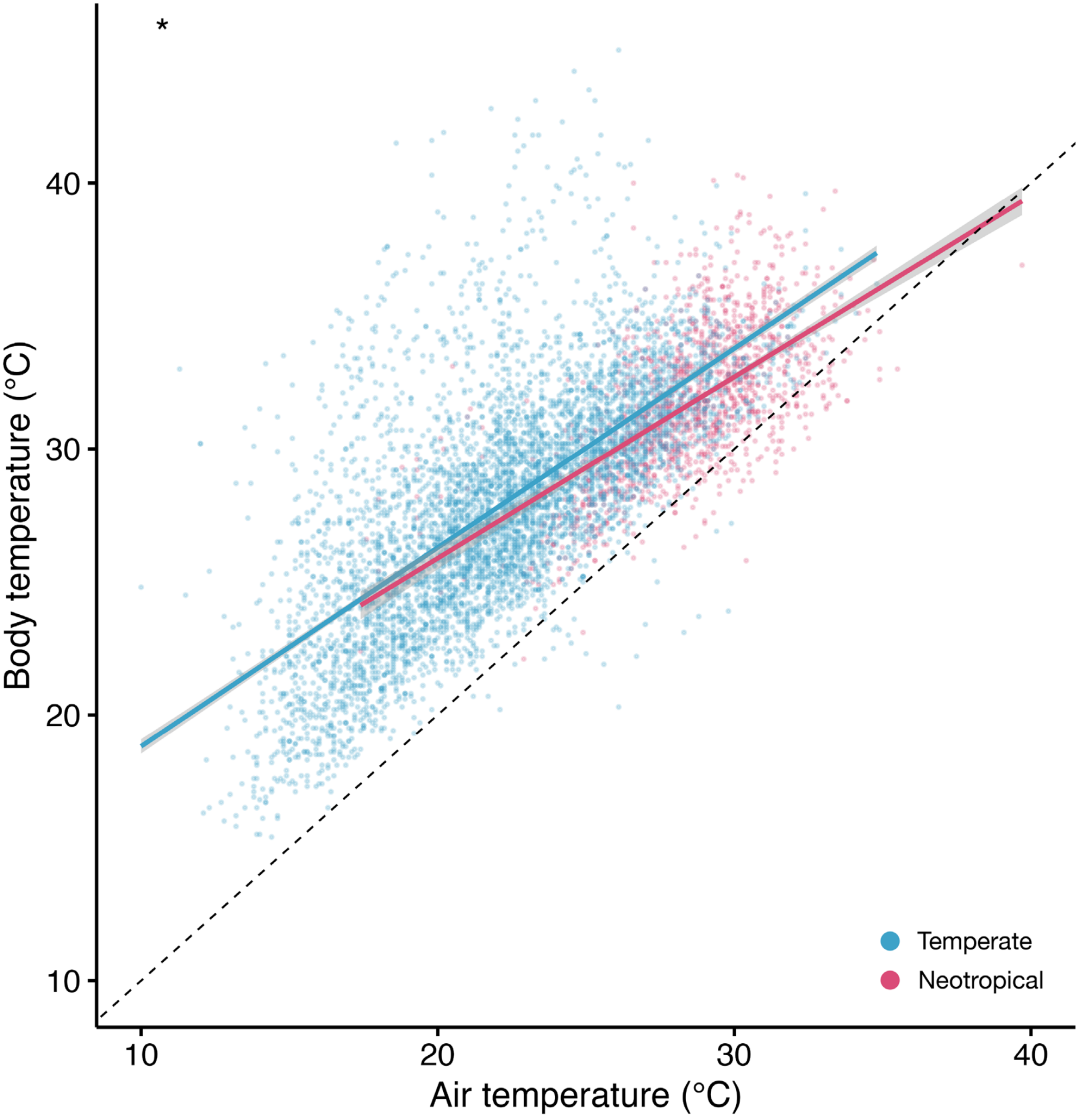
Individual butterfly body temperatures (°C) at different air temperatures (°C) from neotropical (pink) and temperate (blue) regions. Solid lines represent the modelled relationship between body temperature and air temperature. The grey bands show 95% confidence intervals. The black dashed line represents a 1:1 relationship between body and air temperature. The asterisk marks the significant difference in buffering ability estimate between neotropical and temperate butterflies.

Across the whole assemblage, the mean buffering ability of neotropical butterflies (0.350 ± 0.035) was significantly higher than the buffering ability of temperate butterflies (0.220 ± 0.010), with neotropical species appearing better able to reduce their *T*_b_ at higher *T*_a_ (*χ* ^2^ = 14.17, df = 1, *p* < .001; Figure 1). This means that across a 20.0°C range of *T*_a_, predicted neotropical butterfly *T*_b_ would vary by 13.0°C, while predicted temperate butterfly *T*_b_ would vary by 15.6°C. A similar result was obtained when data were restricted to the range of *T*_a_ which occurred in both regions (Figure S3; Results S2).

### 3.2 Do temperate and neotropical butterfly families differ in buffering ability?

**FIGURE 2:**
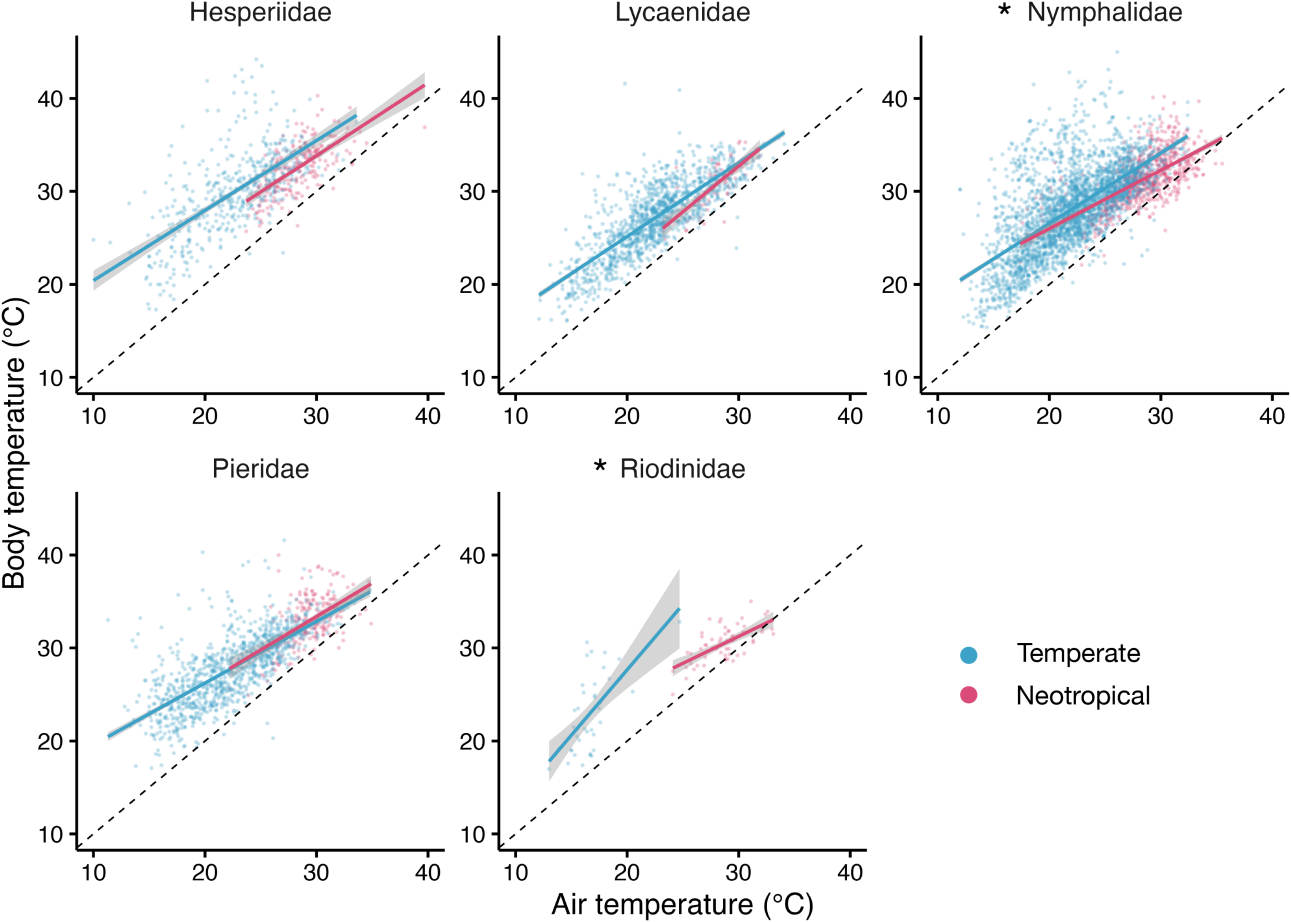
Individual butterfly body temperatures (°C) at different air temperatures (°C) from neotropical (pink) and temperate (blue) regions for each taxonomic family. Solid lines represent the modelled relationship between body temperature and air temperature. The grey bands show the 95% confidence intervals. The black dashed line represents a 1:1 relationship between body and air temperature. Families with a significant difference in buffering ability estimate between neotropical and temperate butterflies are marked with an asterisk.

In the neotropical region, the buffering ability of butterfly families ranged from −0.020 (Lycaenidae) to 0.426 (Pieridae). This means that across a 20.0°C range of *T*_a_, predicted neotropical Lycaenidae *T*_b_ would vary by 20.4°C, while predicted neotropical Pieridae *T*_b_ would vary by 17.2°C. In the temperate region, family‐level buffering ability ranged from −0.404 (Riodinidae) to 0.326 (Pieridae) (Table S6). This means that across a 20.0°C range of *T*_a_, predicted temperate Riodinidae *T*_b_ would vary by 28.1°C, while predicted temperate Pieridae *T*_b_ would vary by 20.2°C. Neotropical Nymphalidae were better at buffering their *T*_b_ against changes in *T*_a_ than Nymphalidae from temperate regions (*χ* ^2^ = 21.66, df = 1, *p* < .001), and Riodinidae from neotropical regions were better at buffering their *T*_b_ against changes in *T*_a_ than *Hamearis lucina*, the only temperate Riodinidae species (*χ* ^2^ = 11.83, df = 1, *p* < .001). This was driven by neotropical Nymphalidae and Riodinidae being better able to lower their *T*_b_ at high *T*_a_ compared to temperate Nymphalidae and Riodinidae (Figure 2). This means that across a 20°C range of *T*_a_, predicted temperate and neotropical Nymphalidae *T*_b_ would vary by 16.4 and 12.1°C, respectively, and predicted temperate and neotropical Riodinidae *T*_b_ would vary by 28.1 and 12.5°C respectively. There were no differences in family‐level buffering ability between temperate and neotropical species for the other three families (Figure 2; Table S6).

When data were restricted to the range of air temperatures which occurred in both regions, a similar result was obtained for Nymphalidae and Pieridae, while Hesperiidae and Lycaenidae showed better buffering ability in temperate than neotropical regions (Figure S4; Table S7). Specifically, temperate Hesperiidae and Lycaenidae increased their *T*_b_ at low *T*_a_ more so than neotropical Hesperiidae and Lycaenidae. It was not possible to model the restricted dataset for Riodinidae, due to limited temperature overlap between regions for the dataset (Results S3).

### 3.3 When controlling for morphology do temperate and neotropical butterflies differ in buffering ability?

Mean forewing length was significantly larger in neotropical butterflies (26.7 ± 0.3 mm) than in temperate butterflies (19.9 ± 0.1 mm) (*F* = 581.75, df = 1, *p* < .001). Neotropical butterflies had a significantly higher mean wing aspect ratio (1.78 ± 0.01) than butterflies from temperate regions (1.71 ± 0.00) (*F* = 233.85, df = 1, *p* < .001). Butterflies from neotropical regions were significantly darker (mean colour scale = 4.12 ± 0.04) than temperate butterflies (mean colour scale = 3.55 ± 0.02) (*F* = 229.43, df = 1, *p* < .001). The best‐fitting model included *T*_a_, region (temperate or neotropical), mean forewing length and mean forewing aspect ratio, and the two‐way interactions between *T*_a_ and each of mean forewing length and mean forewing aspect ratio. This showed that there was no difference in buffering ability between neotropical and temperate butterflies when accounting for differences in morphology (Table 1). Differences in mean forewing length and mean forewing aspect ratio were the main drivers of differences in buffering ability between neotropical and temperate butterflies (Table 1). Butterflies with larger mean forewing lengths and higher mean wing aspect ratios (longer, shallower wings) were better at buffering than their *T*_b_ against changes in *T*_a_ than those with smaller mean forewing lengths and lower mean wing aspect ratios (shorter, deeper wings) (Table 1). The same result was found when the data were restricted to the range of air temperatures which occurred in both regions (Table S8).

**TABLE 1:** Output from the best‐fitting linear mixed effects model, with body temperature (*T*_b_) as the predictor variable and air temperature (*T*_a_), region (neotropical and temperate), mean forewing length, mean forewing aspect ratio and the two‐way interaction between *T*_a_ and each of mean forewing length and mean forewing aspect ratio as response variables.

	Estimate	SE	df	*F*	*p*
Region: temperate	−20.196	4.590	1	22.137	<.001*
Region: tropical	−21.662	0.312			
*T*_a_	1.792	0.156	1	132.181	<.001*
Mean forewing length	6.040	0.903	1	44.770	<.001*
Mean forewing aspect ratio	7.728	2.247	1	11.827	<.001*
*T*_a_: Mean forewing length	−0.456	0.070	1	42.623	<.001*
*T*_a_: Mean forewing aspect ratio	−0.252	0.763	1	10.947	<.001*

*Note*: Mean forewing length was log_10_‐transformed to ensure it was on the same scale as other predictor variables. Species was included as a random effect. Significant terms are marked with an asterisk.

### 3.4 Do temperate and neotropical butterfly assemblages and families differ in their use of microclimate selection and postural thermoregulation, or in their index of thermal specialisation?

**FIGURE 3:**
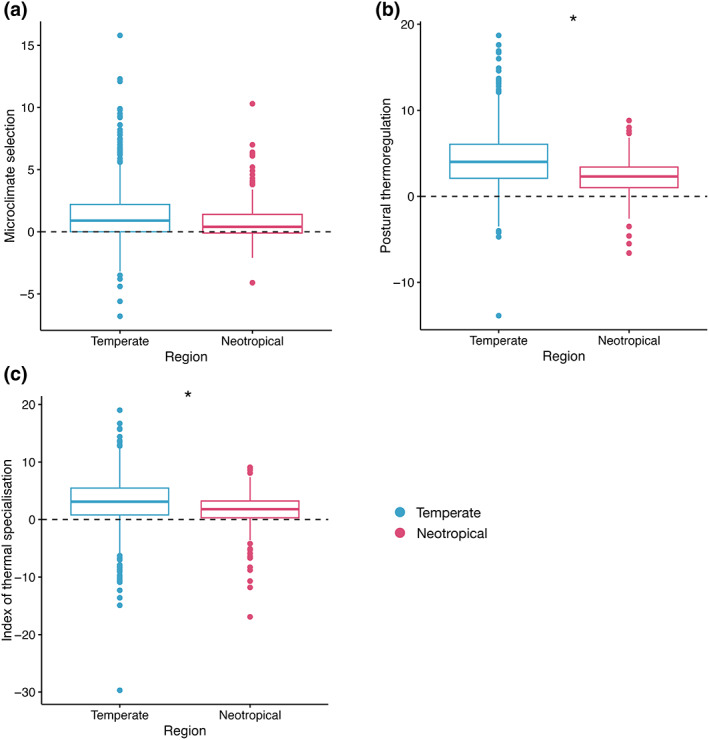
Microclimate selection (the difference between microclimate temperature and air temperature, (a) postural thermoregulation (the difference between body temperature and microclimate temperature, (b) and index of thermal specialisation (postural thermoregulation—microclimate selection, (c) of temperate (blue) and neotropical (pink) butterflies. Significant differences between neotropical and temperate butterflies are marked with an asterisk.

This analysis included a total of 26 temperate (*n* = 919) and 13 neotropical (*n* = 176) species. The use of microclimate selection was not significantly different between temperate and neotropical species (*χ* ^2^ = 0.104, df = 1, *p* = .747) (Figure 3a, Table S9). This result was the same for the two families tested individually (Hesperiidae: *χ* ^2^ = 0.025, df = 1, *p* = .875; Nymphalidae: *χ* ^2^ = 0.007, df = 1, *p* = .932). The use of postural thermoregulation was greater in temperate species than in neotropical (*χ* ^2^ = 8.314, df = 1, *p* = .004), however, when testing families individually only Nymphalidae reflected this result (*χ* ^2^ = 11.86, df = 1, *p* < .001); there was no difference between neotropical and temperate Hesperiidae (*χ* ^2^ = 2.431, df = 1, *p* = .119) (Figure 3b; Table S9). Temperate species used postural thermoregulation to increase their body temperature by a mean of 4.2 ± 0.1°C compared to 2.2 ± 0.2°C in neotropical species. Temperate Nymphalidae used postural thermoregulation to increase their body temperature by a mean of 4.7 ± 0.2°C compared to 2.1 ± 0.2°C in neotropical Nymphalidae. The index of thermal specialisation (ITS) was greater in temperate than neotropical species (*χ* ^2^ = 7.150, df = 1, *p* = .008) and when testing families individually the same was found in Nymphalidae (*χ* ^2^ = 7.970, df = 1, *p* = .005), but not Hesperiidae (*χ* ^2^ = 2.516, df = 1, *p* = .113) (Figure 3c; Table S9). Temperate species ITS was 2.9 ± 0.1°C compared to 1.4 ± 0.3°C in neotropical species and temperate Nymphalidae ITS was 3.3 ± 0.2°C compared to 1.2 ± 0.3°C in neotropical Nymphalidae. There was no effect of air temperature on the difference in how butterflies from neotropical and temperate regions used microclimate selection, postural thermoregulation or ITS, across the whole assemblage and between the two families tested individually (Hesperiidae and Nymphalidae) (Table S9).

When data were restricted to the range of air temperatures which occurred in both regions, there was no difference in the use of microclimate selection, postural thermoregulation or ITS between butterflies from neotropical and temperate regions (Results S4; Figure S5 and Table S10). Microclimate selection and ITS did not differ significantly between neotropical and temperate Nymphalidae, but temperate Nymphalidae used postural thermoregulation more than neotropical Nymphalidae (Results S4; Figure S5 and Table S10). There were insufficient data to undertake this analysis for the other families.

## 4 DISCUSSION

We found that at the assemblage level, neotropical butterflies are better at buffering their body temperature against changes in air temperature than temperate butterflies. At the family level the same trend was found in Nymphalidae and in the comparison of the three neotropical Riodinidae species to the single European species, but there was no difference for Lycaenidae, Pieridae or Hesperiidae. In the dataset restricted to the range of air temperatures overlapping in both regions, temperate Hesperiidae and Lycaenidae showed better buffering ability than their neotropical counterparts. When controlling for morphological characteristics, there was no difference in buffering ability between the two regions, but larger species and those with higher wing aspect ratios (longer, shallower wings) were better at buffering their body temperature against changes in air temperature than smaller species and those with lower wing aspect ratios (shorter, deeper wings). There were no differences in the use of microclimate selection for thermoregulation between butterflies from neotropical and temperate regions at the assemblage or family level. At the assemblage level, postural thermoregulation was used more to control temperature by temperate than neotropical species, and this was also true for Nymphalidae, but not for Hesperiidae, at the family level. Overall, the index of thermal specialisation was greater in temperate butterflies than in neotropical butterflies, but at the family level this was only the case for Nymphalidae and not Hesperiidae.

### 4.1 Thermal buffering ability

Across the whole assemblage, neotropical butterflies had a better buffering ability than temperate butterflies, with neotropical species having relatively cooler body temperatures at higher air temperatures than temperate species. At the family level, only Nymphalidae and Riodinidae showed a difference in buffering ability between neotropical and temperate regions, with neotropical Nymphalidae and Riodinidae species being better at buffering compared to their temperate counterparts. However, as there was only one temperate and three neotropical Riodinidae species in our dataset, it is not possible to make general assumptions about the thermoregulatory capacities of butterflies from this family, and our finding likely reflects the poor buffering ability of *Hamearis lucina*, the only European species. As half of the species sampled in both temperate and neotropical regions were from the Nymphalidae family (47 of 91 species) it is likely that this family was driving the trend observed at the assemblage level. Notably, this result was the same when data were restricted to the range of air temperatures which occurred in both regions, indicating that this is a pattern which is independent of the specific range of temperatures butterflies experienced during this study. However, in the restricted dataset, temperate Hesperiidae and Lycaenidae species showed better buffering ability than their neotropical counterparts, suggesting that they were better able to cope with changes in air temperature. This result was likely driven by temperate butterflies warming up more at a given air temperature, in an effort to increase activity in the cooler, temperate environment.

The buffering ability response from the dataset including the full range of air temperatures was driven by neotropical species cooling down more than temperate species at high air temperatures. Indeed, at the species level, some neotropical species maintained their body temperature at temperatures lower than air temperature, when temperatures were high (e.g. *Heliconius hecale melicerta*). There is likely to be strong selection among neotropical species to avoid high temperatures, as tropical species live closer to their thermal safety margin (Deutsch et al., 2008; Sunday et al., 2012, 2014, 2019). The better buffering ability of neotropical butterflies may be further explained by differences in habitat type between the neotropical and temperate regions. There was more tree cover and shade in the neotropical sites compared to the temperate sites, and hence a greater availability of cool microclimates. At high air temperatures, neotropical butterflies may use these marginally cooler microclimates to lower their body temperature, avoiding their thermal limit and the associated decrease in survival and fitness.

It is important to note that our analysis only included data for 54 of 601 neotropical species found in the region (Basset et al., 2015), and 40 of 496 temperate species (Wiemers et al., 2018). This means our results may not be representative of the entire tropical and temperate butterfly assemblages found in the wider regions. However, the neotropical species we sampled are not endemic to Panama and are found elsewhere in the neotropics, making our tropical data likely to be a reliable subsample of the wider neotropical butterfly community. It is also possible that the species we surveyed have a higher than average thermoregulatory ability for their assemblage, as butterflies which thermoregulate efficiently are able to be more active, hence are more likely to see seen and sampled. Nonetheless, we sampled a large pool of species at random, which is likely to reduce the chance of this bias affecting results, and any bias for abundant or conspicuous species is likely to be similar in both regions. Finally, the differences in buffering ability we recorded between regions could be the result of evolutionary history and genetic drift, rather than selection pressures, as neotropical and temperate butterflies diverged a long time ago. However, by undertaking family‐level analysis we partly control for phylogeny‐driven differences in buffering ability. Additionally, thermoregulation is a highly important factor in butterfly ecology, so traits related to temperature control are likely to be under strong selection pressure.

### 4.2 The importance of morphological traits

Alongside the better buffering ability of neotropical species at the assemblage level, we found that neotropical butterflies are generally larger and darker and have a higher wing aspect ratio (longer, shallower wings) than temperate butterflies. Larger species and those with higher wing aspect ratios had a better buffering ability than smaller species and those with lower wing aspect ratios (shorter, deeper wings). This reflects similar results elsewhere. For example, large temperate butterflies buffer air temperature changes better than small butterflies (Bladon et al., 2020), because their larger wing surface area allows them to better regulate their temperature by basking (Kingsolver, 1988; Shanks et al., 2015; Wasserthal, 1975; Xing et al., 2016; Zeuss et al., 2014). Additionally, large butterflies may be more mobile and able to search out cooler microclimates, allowing them to buffer against high air temperatures (Chazot et al., 2016). High wing aspect ratio is associated with greater mobility and flying speed (Hassall, 2015). As far as we are aware it is unknown how wing aspect ratio impacts thermoregulatory ability, however, greater mobility may enable neotropical butterflies to search out favourable microclimates more effectively. Therefore, the better buffering ability of neotropical butterflies compared to temperate butterflies in our dataset could be due to either the larger wing length or higher wing aspect ratio of the neotropical species. However, when these effects were controlled for, the difference in buffering ability between regions did not persist.

### 4.3 Microclimate selection, postural thermoregulation and index of thermal specialisation

We found that there was no difference in the use of thermoregulation via microclimate selection between neotropical and temperate species, but temperate species used postural thermoregulation to raise their body temperature more than neotropical species. Neotropical species generally maintained their body temperature closer to ambient air temperature than temperate species. Using postural thermoregulation to heat up is likely to be an adaptation to cooler climates. Butterflies require heat to become active, and loose heat as they fly (Advani et al., 2019). When there is a large difference between air temperature and optimal body temperature, as in temperate systems, heat loss during flight is greater, and so temperate species may be under stronger selection than neotropical species to use postural thermoregulation to heat up, enabling them to become active and increase flight duration in cooler air temperatures. Indeed, temperate butterflies from higher latitudes take‐off at higher body temperatures than butterflies from lower latitudes (Advani et al., 2019). For temperate species in our study, undertaking postural thermoregulation to gain more heat before take‐off might compensate for the additional heat lost during flight at lower air temperatures, which could increase activity and survival. This result could also be due to other morphological differences between neotropical and temperate butterflies, such as colour, with lighter species, which were found more commonly in the temperate than neotropical study sites, being able to undertake postural thermoregulation better than darker species. Alternatively, it may be a consequence of the different habitat types that were sampled between neotropical and temperate regions. In the temperate region more open habitats were sampled, which would have provided greater opportunities for basking and could explain why temperate butterflies undertook postural thermoregulation more than neotropical butterflies.

We found that neotropical species were more thermally specialist, relying relatively more on the selection of available microclimates than postural thermoregulation, when compared to temperate species. However, this result is driven by temperate species undertaking postural thermoregulation to increase their body temperature. Neotropical species maintained their body temperature closer to ambient temperature than temperate species did, especially at higher air temperatures, presumably as a mechanism to avoid overheating. This could make them vulnerable to extreme heatwave events, as, if air temperature increases, their ability to avoid temperatures outside their thermal safety margin might become limited.

At the family level, we found the same result in Nymphalidae, the most abundant family in our dataset, but not Hesperiidae, so Nymphalidae were likely driving the assemblage‐level trend. Nymphalidae species are often larger than those in other butterfly families, and it is possible that their wing size enables them to undertake postural thermoregulation more effectively, as larger wings can better control temperature through basking than smaller wings.

### 4.4 Implications

Climate change, through increasing temperatures, is expected to negatively affect ectotherms, decreasing their performance and, in extreme cases, resulting in extinction (Diamond & Yilmaz, 2018; Johansson et al., 2020; Mi et al., 2022). By buffering their body temperature against changes in air temperature using a combination of microclimate selection and postural thermoregulation, butterflies might be able to mitigate some of these negative effects. We found that neotropical butterfly species, which may be living closer to their thermal limit than temperature species, buffer their body temperature against changes in air temperature better than temperate species, primarily by cooling down more at higher air temperatures (Deutsch et al., 2008; Sunday et al., 2012, 2014, 2019). In the restricted dataset, temperate Hesperiidae and Lycaenidae were better at buffering their body temperature against changes in air temperature than their neotropical counterparts, but this was driven by their ability to warm up more at a given air temperature. Similarly, temperate butterflies, and in particular Nymphalidae, were better at using behaviour for thermoregulation than neotropical butterflies, but this is likely to be an adaptation for warming up in cool temperate climates, and may not indicate a good ability to cool down under rising temperatures. Therefore, both neotropical and temperate butterfly species show signs of vulnerability to rising temperatures, with no overriding ecological difference in sensitivity to climate change between bioclimatic regions. However, the degree to which physiological acclimation or genetic adaptation could offset the negative effects of increasing temperatures on these butterflies remains unclear (Diamond & Yilmaz, 2018; Seebacher et al., 2015; Sgrò et al., 2016; Sheldon & Tewksbury, 2014).

Our study has focused solely on the adult stage, but thermal sensitivity and hence vulnerability to changing temperatures varies markedly between life stages (Kingsolver et al., 2011). Indeed, life stage is the most important factor for predicting tolerance to changes in temperature, and mortality mostly occurs before the adult stage (Bowler & Terblanche, 2008). Eggs are thought to be the most vulnerable life stage, compared to larvae, pupae and adults, as a consequence of their physiology and their immobility, which means they cannot locate more favourable microclimates (Kingsolver et al., 2011). Larvae are also likely to be sensitive to increasing temperatures, as although mobile, their dispersal ability is limited (Kingsolver et al., 2011). Additionally, most Lepidoptera spend more time in the larval than the adult stage, meaning temperature changes are more likely to impact this life stage. Therefore, future studies should investigate the buffering ability and thermoregulatory mechanisms of all butterfly life stages.

Although microclimates may offer thermal refugia for adult Lepidoptera, the extent to which they will benefit less mobile life stages with regards to temperature control and coping with temperature extremes remains unclear (Montejo‐Kovacevich et al., 2020). Habitat degradation and land‐use change has been shown to decrease the availability of cooler microclimates and hence the temperature buffering potential of a habitat (Jucker et al., 2020). As conversion of land for agricultural use continues to accelerate and global temperatures rise, the protection of pristine habitats for their temperature buffering potential may become increasingly important.

## 5 CONCLUSION

To our knowledge, this is the first study comparing the thermal buffering ability of ectotherms between tropical and temperate systems. By using standardised methods to measure buffering ability and mechanisms, we provide a template for making cross‐latitudinal comparisons within taxa, increasing understanding of how species across latitudes cope with changing temperatures. We stress the need for further cross‐latitudinal studies comparing the thermal capabilities of tropical and temperate species. Despite over half of the world's insect species living in tropical systems, most research focuses on the thermal capabilities of temperate ectotherms (Garcia‐Robledo et al., 2020; Stork, 2018). Future studies should focus on tropical ectotherms and expand knowledge of how these species regulate temperature and their potential vulnerabilities to climate change. We also highlight the importance of considering species' morphological traits, and use of microclimate selection and postural thermoregulation when predicting the vulnerability of tropical and temperate species to future climate change. Future studies should also investigate the role of habitat structure in the buffering ability of butterflies, for example determining how favourable microclimates may act as refuges from extreme temperatures for tropical and temperate species, paving the way to informing management options that could protect species from warming. Most importantly, our results show that individual species have their own individual thermoregulatory capabilities. We stress the need for caution when making generalised assumptions of species' vulnerability to climate change based solely on the thermal environment they inhabit.

## AUTHOR CONTRIBUTIONS

The study was designed by Andrew J. Bladon, Edgar C. Turner, Benita C. Laird‐Hopkins and Esme Ashe‐Jepson. Data were collected by Benita C. Laird‐Hopkins, Esme Ashe‐Jepson, Irena Kleckova, Andrew J. Bladon, Gwen E. Hitchcock, Josh Hellon, Ana Cecilia Zamora, Amanda F. Savage, Alex McFarlane, Stephany Arizala Cobo, Daniel Linke, Inga Freiberga and Lucy Eberhardt. Benita C. Laird‐Hopkins conducted the analyses and wrote the manuscript. All authors contributed substantially to revisions.

## CONFLICT OF INTEREST STATEMENT

The authors declare no potential conflict of interest.

## Supporting information


Data S1.

## Data Availability

The data that support the findings of this study are openly available in Zenodo at http://doi.org/10.5281/zenodo.7900256.
