# Supplementary material for: Thermoregulatory ability and mechanism do not differ consistently between neotropical and temperate butterflies
Source: Glob Chang Biol. 2023 Jun 14;29(15):4180–92. doi: 10.1111/gcb.16797 (PMC10946725; doi:10.1111/gcb.16797)
Supplement: Supplementary file 1 — Data S1. [file GCB-29-4180-s001.pdf]

Thermoregulatory ability and mechanism does not differ consistently between neotropical and temperate butterflies.

Supplementary material.

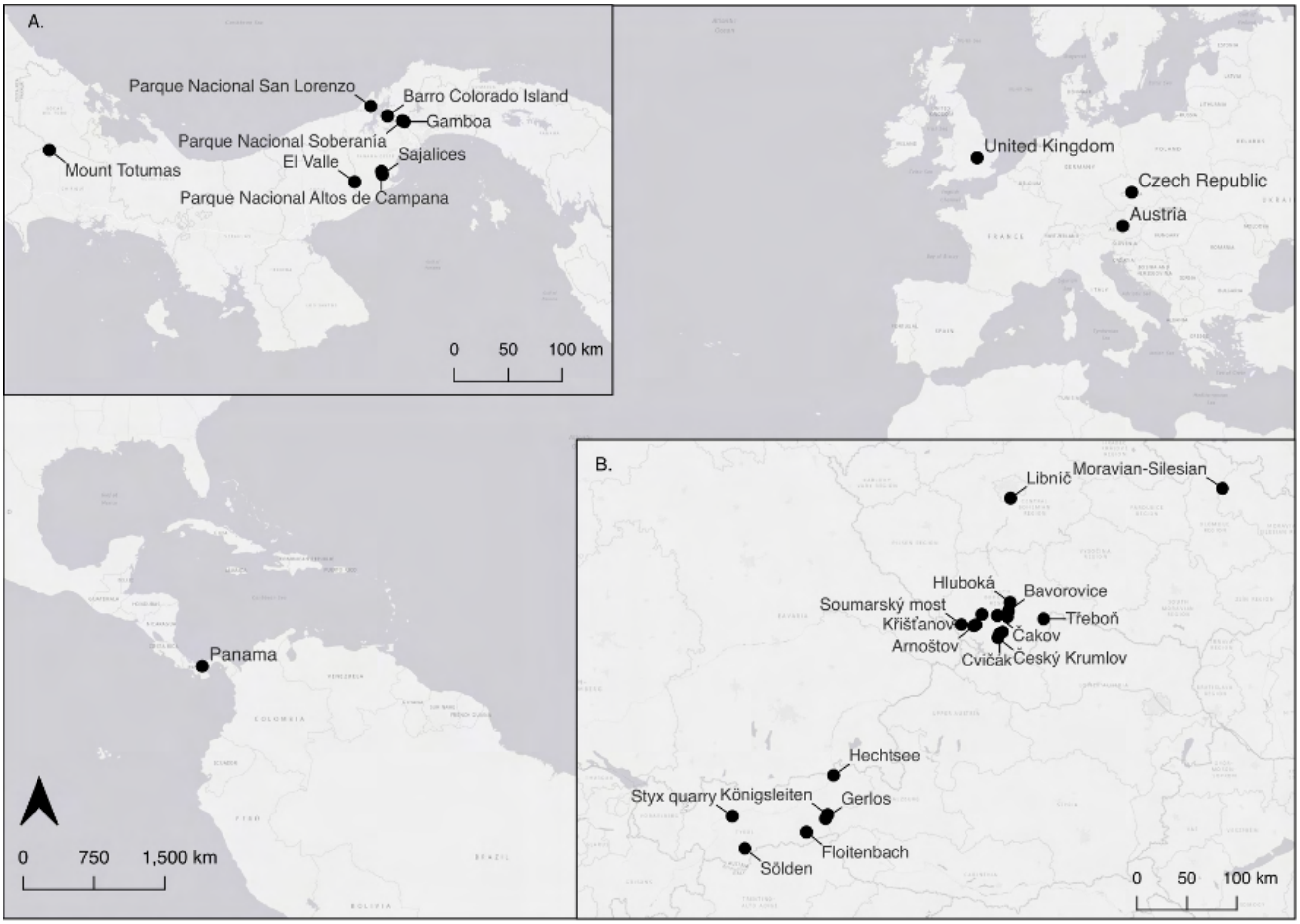

SUPPLEMENTARY FIGURE 1. *Broad study locations in Panama, Austria, the Czech Republic and the UK (main figure) with insets of field sites in Panama (neotropical, inset A) and Austria and the Czech Republic (temperate, inset B) (Supplementary Table 1) (ESRI, 2022). See Bladon et al. 2020 for the location of UK field sites.*

SUPPLEMENTARY TABLE 1: *Study sites in Austria, the Czech Republic, the United Kingdom and Panama, including coordinates, elevation (meters above sea level) and land type.*

Site	Coordinates		Elevation	Land type
	N	E		
TEMPERATE Austria				
Floitenbach	47.099153	11.810562	996	Exposed ground, alpine grassland meadows, encroaching scrub and secondary forest
Gerlos	47.218297	12.062329	1346	Exposed ground, alpine grassland meadows and forest edge
Hechtsee	47.599623	12.164717	670	Forest edge
Königsleiten	47.253974	12.089779	2050	High alpine grassland meadow
Sölden	46.954529	11.008711	1511	Exposed ground, alpine grassland meadows, encroaching scrub and secondary forest
Styx quarry	47.239532	10.844794	733	Exposed ground, alpine grassland meadows, forest edge and secondary forest
TEMPERATE Czech Republic				
Arnoštov	48.894660	13.994615	840	Grassland meadows
Bavorovice	49.023401	14.443310	375	Grassland meadows
Čakov	48.984240	14.296788	426	Grassland meadows
České Budějovice	48.974414	14.430564	442	Grassland meadows and secondary forest edge
Český Krumlov	48.793907	14.304518	503	Grassland meadows, roadside verges, secondary forest
Cvičák	48.829148	14.326293	591	Grassland meadows, encroaching scrub and marshland
Hluboká	49.094034	14.465941	405	Grassland meadows and secondary forest
Křišťanov	48.906300	14.019557	940	Grassland meadows
Libníč	49.014427	14.547689	333	Grassland meadows
Moravian-Silesian	50.056424	17.228311	1444	Grassland meadows
Rájov	48.843466	14.365909	459	Grassland meadows
Soumarský most	48.907138	13.827730	754	Grassland meadows and secondary forest
Třeboň	48.955848	14.900563	446	Grassland meadows
Zelený	48.993960	14.096682	805	Grassland meadows
TEMPERATE United Kingdom				
Ben Lawers	56.532870	-4.273260	614	Montane grassland
Blows Downs	51.883210	-0.495800	202	Calcareous grassland meadows and scrub
Haweswater	54.507560	-2.845980	652	Montane grassland
Irton Fell	54.406720	-3.340000	236	Montane grassland

Pegsdon Hills	51.953540	-0.370200	100	Calcareous grassland meadows and scrub
Totternhoe Knolls	51.889890	-0.580390	134	Secondary calcareous grassland meadows and scrub
Totternhoe Quarry	51.891990	-0.568360	125	Secondary calcareous grassland meadows and scrub
Winterbourne Downs	51.149630	-1.685000	114	Secondary calcareous grassland meadows and scrub
NEOTROPICAL Panama				
Barro Colorado Island	9.164550	-79.837554	40	Secondary semi-deciduous lowland tropical wet forest
El Valle	8.617975	-80.115683	735	Lowland tropical wet encroaching scrub
Gamboa	9.116637	-79.696492	50	Lowland managed urban green spaces
Mount Totumas	8.882948	-82.683699	1880	Lower mountain rain forest and managed agroforestry sites
Parque Nacional Altos de Campana	8.705518	-79.887839	284	Premontane wet encroaching scrub and secondary forest
Parque Nacional San Lorenzo	9.246993	-79.978955	177	Secondary lowland tropical wet forest
Parque Nacional Soberanía	9.125578	-79.714579	84	Secondary semi-deciduous lowland tropical wet forest
Sajalices	8.680064	-79.880754	98	Lowland tropical wet encroaching scrub and secondary forest

SUPPLEMENTARY TABLE 2. *Sources of photos used to calculate wing aspect ratio.*

Name of organisation	Website
Butterflies of America	www.butterfliesofamerica.com
Natural History Museum Entomology collection	www.nhm.ac.uk/our-science/collections/entomology-collections.html
BOLD systems	www.boldsystems.org
MCZbase, Museum of Comparative Zoology, Harvard University	https://mczbase.mcz.harvard.edu
FUNDus, Universität Hamburg	https://fundus.uni-hamburg.de
Mapování motýlů ČR	www.lepidoptera.cz
Finnish Biodiversity Information Facility	https://laji.fi
Farfalle Italiane	www.farfalleitalia.it
Svenska fjärilar	www3.nrm.se/en/svenska_fjarilar/l/lepidoptera_family.html
Biological Library	www.biolib.cz

SUPPLEMENTARY PANEL 1. Pearson correlation coefficient between predictor variables used for the linear mixed effect model in analysis 2: air temperature (T_a), mean forewing length (mm), mean wing aspect ratio and wing colour.

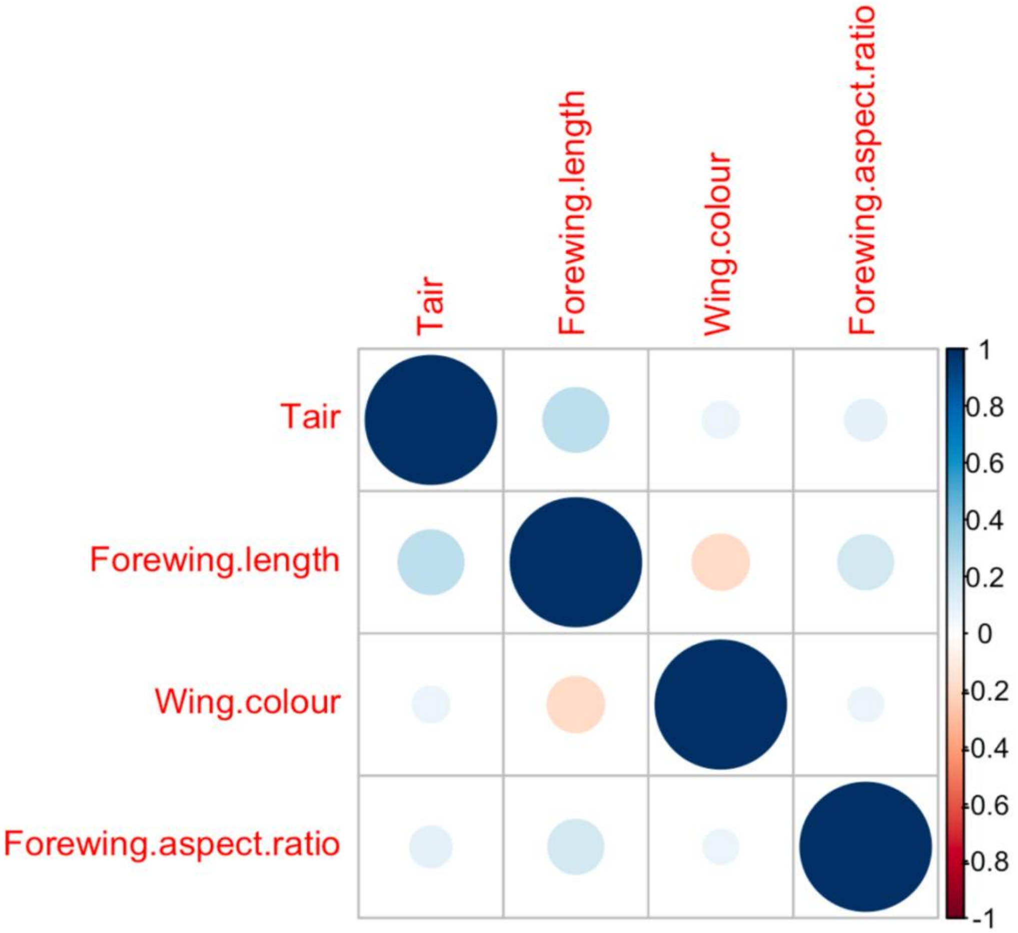

SUPPLEMENTARY TABLE 3. *Results of one-way ANOVAs with mean forewing length (mm), mean wing aspect ratio and colour fitted seperately as the response variable and family as the predictor variable. Statistically significant p-values are marked with an asterisk*

	df	F	*p*
Mean forewing length	5	891	<0.001*
Mean wing aspect ratio	5	149	<0.001*
Colour	5	6633	<0.001*

SUPPLEMENTARY TABLE 4. *Temperate and neotropical species, with their taxonomic family, mean forewing length (mm), mean forewing aspect ratio, wing colour, the sample size of individuals we obtained in the field, buffering ability estimate (calculated as the slope of the regression of thoracic temperature against air temperature, subtracted from 1, following Bladon et al 2020) and whether or not we have ≥10 microclimate temperature data points per species. If ≥10 microclimate temperature data points were available for a species, this species was included in the analysis of microclimate selection, postural thermoregulation and index of thermal specialisation.*

Species	Family	Forewing length mm	Forewing aspect ratio	Wing colour	Sample size	Buffering ability estimate	Microclimate data
TEMPERATE							
Aglais io	Nymphalidae	28.479	1.635	4	83	0.362	Y
Aglais urticae	Nymphalidae	24.618	1.727	3	42	0.295	Y
Anthocharis cardamines	Pieridae	21.124	1.776	2	54	0.601	N
Aphantopus hyperantus	Nymphalidae	21.550	1.631	5	353	0.212	Y
Araschnia levana	Nymphalidae	17.500	1.858	5	124	0.164	Y
Argynnis paphia	Nymphalidae	32.000	1.785	3	33	0.223	Y
Aricia agestis	Lycaenidae	13.246	1.770	4	152	0.191	Y
Carterocephalus palaemon	Hesperiidae	14.000	2.069	4	10	0.537	Y
Celastrina argiolus	Lycaenidae	15.675	1.601	3.5	24	0.311	N
Coenonympha pamphilus	Nymphalidae	15.063	1.641	4	330	0.133	N
Colias hyale	Pieridae	25.000	1.754	2	15	0.080	N
Cupido minimus	Lycaenidae	11.838	1.696	4	128	0.189	N
Erebia aethiops	Nymphalidae	23.000	1.741	4	115	0.290	Y
Erebia epiphron	Nymphalidae	16.362	1.831	4	239	-0.100	Y
Erebia euryale	Nymphalidae	21.500	1.688	4	59	0.218	Y
Erebia ligea	Nymphalidae	25.500	1.694	4	175	0.415	Y
Erebia medusa	Nymphalidae	21.500	2.017	4	22	0.675	N
Erebia melampus	Nymphalidae	14.000	1.679	4	19	0.214	N
Erebia pandrose	Nymphalidae	21.000	1.764	4	43	0.472	N
Erynnis tages	Hesperiidae	14.592	1.738	5	78	0.213	N
Gonepteryx rhamni	Pieridae	29.279	1.598	1.5	120	0.393	Y
Hamearis lucina	Riodinidae	15.062	1.726	4	40	-0.404	N
Leptidea sinapis	Pieridae	21.500	2.017	1	74	0.190	N
Lycaena phlaeas	Lycaenidae	14.717	1.699	4	50	0.128	Y
Lysandra coridon	Lycaenidae	17.084	1.699	4	211	0.221	Y
Maniola jurtina	Nymphalidae	22.665	1.553	4	467	0.201	Y
Melanargia galathea	Nymphalidae	24.382	1.662	3.5	267	0.120	Y

Ochlodes sylvanus	Hesperiidae	15.748	1.873	4	141	0.455	Y
Pararge aegeria	Nymphalidae	21.478	1.643	4	173	0.195	Y
Pieris brassicae	Pieridae	30.494	1.768	1	128	0.370	Y
Pieris napi	Pieridae	23.502	1.730	1	311	0.273	Y
Pieris rapae	Pieridae	23.943	1.743	1	278	0.317	Y
Polygonia c-album	Nymphalidae	23.580	1.647	4	42	0.135	Y
Polyommatus icarus	Lycaenidae	14.422	1.718	4	640	0.192	Y
Pyronia tithonus	Nymphalidae	18.993	1.592	4	141	0.103	Y
Speyeria aglaja	Nymphalidae	28.944	1.652	3	20	0.040	N
Thymelicus lineola	Hesperiidae	12.811	1.799	4	72	0.334	Y
Thymelicus sylvestris	Hesperiidae	13.839	1.884	4	153	0.135	Y
Vanessa atalanta	Nymphalidae	30.270	1.643	4.5	20	0.487	N
Vanessa cardui	Nymphalidae	29.161	1.769	4	36	0.155	N
NEOTROPICAL							
Adelpha cytherea	Nymphalidae	24.335	1.551	4	24	0.364	N
Aides dysoni	Hesperiidae	24.814	2.233	5	14	-0.211	N
Anartia fatima	Nymphalidae	28.902	1.658	5	63	0.340	Y
Anartia jatrophae	Nymphalidae	28.705	1.729	3.5	27	-0.113	N
Anthanassa tulcis	Nymphalidae	15.503	1.820	5	30	0.298	Y
Aphrissa statira	Pieridae	32.204	1.584	2	24	0.437	N
Arawacus togarna	Lycaenidae	14.492	1.463	3.5	13	0.157	N
Battus polydamas	Papilionidae	43.615	1.900	6	15	0.183	N
Burnsius orcus	Hesperiidae	14.648	1.848	3.5	26	0.230	Y
Calephelis sp	Riodinidae	11.056	1.575	4	27	0.313	Y
Cecropterus dorantes	Hesperiidae	23.392	1.804	5	13	0.064	N
Chlosyne lacinia	Nymphalidae	22.347	1.847	4.5	16	0.091	N
Cissia pompilia	Nymphalidae	18.264	1.641	5	18	-0.099	Y
Cissia terrestris	Nymphalidae	15.592	1.572	5	13	0.460	N
Cupido comyntas	Lycaenidae	10.200	1.615	4	12	-0.092	N
Danaus plexippus	Nymphalidae	47.315	1.966	4.5	22	0.547	N
Detritivora hermodora	Riodinidae	11.211	1.608	5	14	0.452	N
Dione juno	Nymphalidae	37.611	2.090	3	20	0.366	N
Dione moneta	Nymphalidae	36.991	2.013	3	12	0.676	N
Dryas iulia	Nymphalidae	41.658	2.471	3	36	0.286	N
Dynamine paulina	Nymphalidae	21.453	1.581	4	17	0.141	N

Eurema albula	Pieridae	18.411	1.549	1	22	0.400	N
Eurema daira	Pieridae	16.092	1.939	1.5	28	0.166	N
Heliconius doris	Nymphalidae	39.581	2.270	6	42	0.408	N
Heliconius erato demophoon	Nymphalidae	33.534	1.972	6	65	0.626	N
Heliconius hecale melicerta	Nymphalidae	42.244	2.082	4.5	44	0.533	N
Heliconius sara	Nymphalidae	32.750	2.260	6	35	0.601	N
Hemiargus hanno	Lycaenidae	9.169	1.692	4	42	-0.317	N
Hermeuptychia hermes	Nymphalidae	16.091	1.497	5	47	-0.055	Y
Hylephila phyleus	Hesperiidae	14.823	2.000	4	16	0.686	N
Itaballia demophile	Pieridae	28.091	1.637	1	34	0.211	N
Itaballia pandosia	Pieridae	21.062	1.648	1	13	0.072	N
Janatella leucodesma	Nymphalidae	18.343	1.712	3.5	35	0.310	Y
Juditha caucana	Riodinidae	15.768	1.642	3.5	18	0.362	N
Junonia evarete zonalis	Nymphalidae	26.759	1.584	4	50	0.498	Y
Magneuptychia libye	Nymphalidae	24.783	1.532	5	13	0.162	N
Marpesia chiron	Nymphalidae	30.094	1.792	5	10	0.786	N
Melinaea idae	Nymphalidae	43.445	2.109	4.5	10	0.511	N
Morpho helenor	Nymphalidae	58.428	1.593	3	25	0.296	N
Morpho menelaus	Nymphalidae	68.612	1.763	3	13	0.648	N
Nica flavilla	Nymphalidae	20.523	1.594	4	28	-0.149	Y
Pareuptychia ocirrhoe	Nymphalidae	18.896	1.549	3.5	12	0.030	N
Parides eurimedes	Papilionidae	38.662	1.887	6	13	0.553	N
Parides sesostris	Papilionidae	39.950	1.873	6	24	0.367	N
Phoebis argante	Pieridae	33.340	1.473	2	39	1.005	N
Phoebis philea	Pieridae	38.473	1.497	2	12	0.337	N
Phoebis sennae	Pieridae	33.151	1.518	2	37	0.661	N
Pompeius pompeius	Hesperiidae	14.631	1.835	5	36	0.170	N
Spicauda procne	Hesperiidae	22.182	1.822	5	29	0.423	Y
Spicauda simplicius	Hesperiidae	22.284	1.654	5	27	-0.255	N
Staphylus ascalaphus	Hesperiidae	13.008	1.727	5.5	15	0.102	Y
Staphylus azteca	Hesperiidae	12.760	1.768	5	10	0.413	N
Staphylus vulgata	Hesperiidae	12.950	1.765	5	12	0.360	Y
Vehilius stictomenes	Hesperiidae	11.425	1.902	5	21	0.090	Y

SUPPLEMENTARY RESULTS 1. Summary statistics of the three temperate regions.

In the UK, 1,884 individuals were measured across 29 species and five families (Hesperiidae, Lycaenidae, Nymphalidae, Pieridae and Riodinidae). T_a ranged from 10.0 °C to 34.8 °C, with a mean of 22.3 °C. T_b ranged from 15.4 °C to 45.0 °C, with a mean of 28.1 °C. In Austria, 383 individuals were measured across six species and one family (Nymphalidae). T_a ranged from 16.2 °C to 29.4 °C, with a mean of 22.4 °C. T_b ranged from 20.7 °C to 34.7 °C, with a mean of 27.5 °C. In the Czech Republic 1,302 individuals were measured across 33 species and four families (Hesperiidae, Lycaenidae, Nymphalidae and Pieridae). T_a ranged from 14.4 °C to 32.2 °C, with a mean of 22.0 °C. T_b ranged from 16.5 °C to 39.4 °C, with a mean of 27.8 °C.

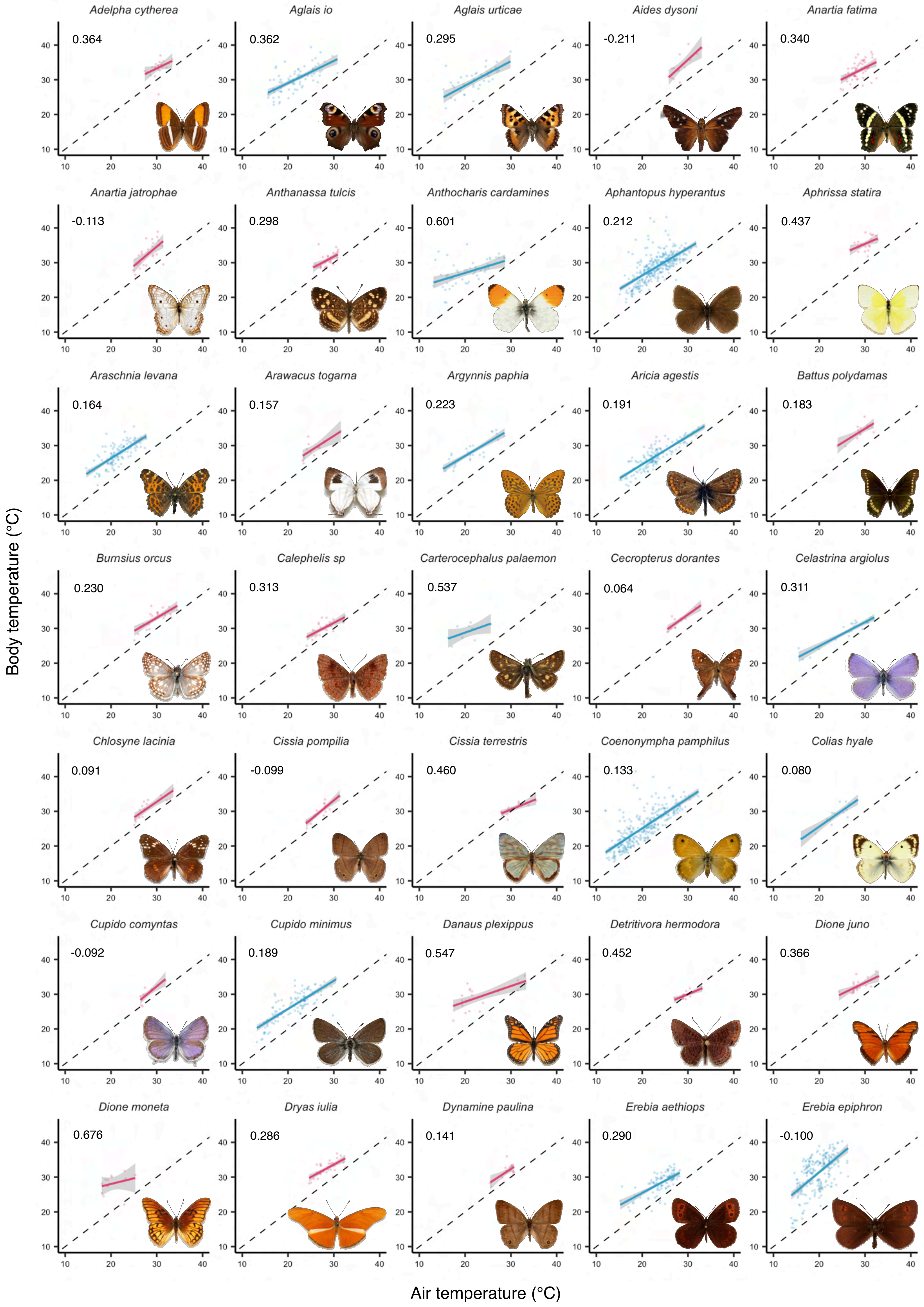
Adelpha cytherea
0.364
Aglais io
0.362
Aglais urticae
0.295
Aides dysoni
-0.211
Anartia fatima
0.340
Anartia jatrophae
-0.113
Anthanassa tulcis
0.298
Anthocharis cardamines
0.601
Aphantopus hyperantus
0.212
Aphrissa statira
0.437
Araschnia levana
0.164
Arawacus togarna
0.157
Argynnis paphia
0.223
Aricia agestis
0.191
Battus polydamas
0.183
Burnsius orcus
0.230
Calephelis sp
0.313
Carterocephalus palaemon
0.537
Cecropterus dorantes
0.064
Celastrina argiolus
0.311
Chlosyne lacinia
0.091
Cissia pompilia
-0.099
Cissia terrestris
0.460
Coenonympha pamphilus
0.133
Colias hyale
0.080
Cupido comyntas
-0.092
Cupido minimus
0.189
Danaus plexippus
0.547
Detritivora hermodora
0.452
Dione juno
0.366
Dione moneta
0.676
Dryas iulia
0.286
Dynamine paulina
0.141
Erebia aethiops
0.290
Erebia epiphron
-0.100
Body temperature (°C)
Air temperature (°C)

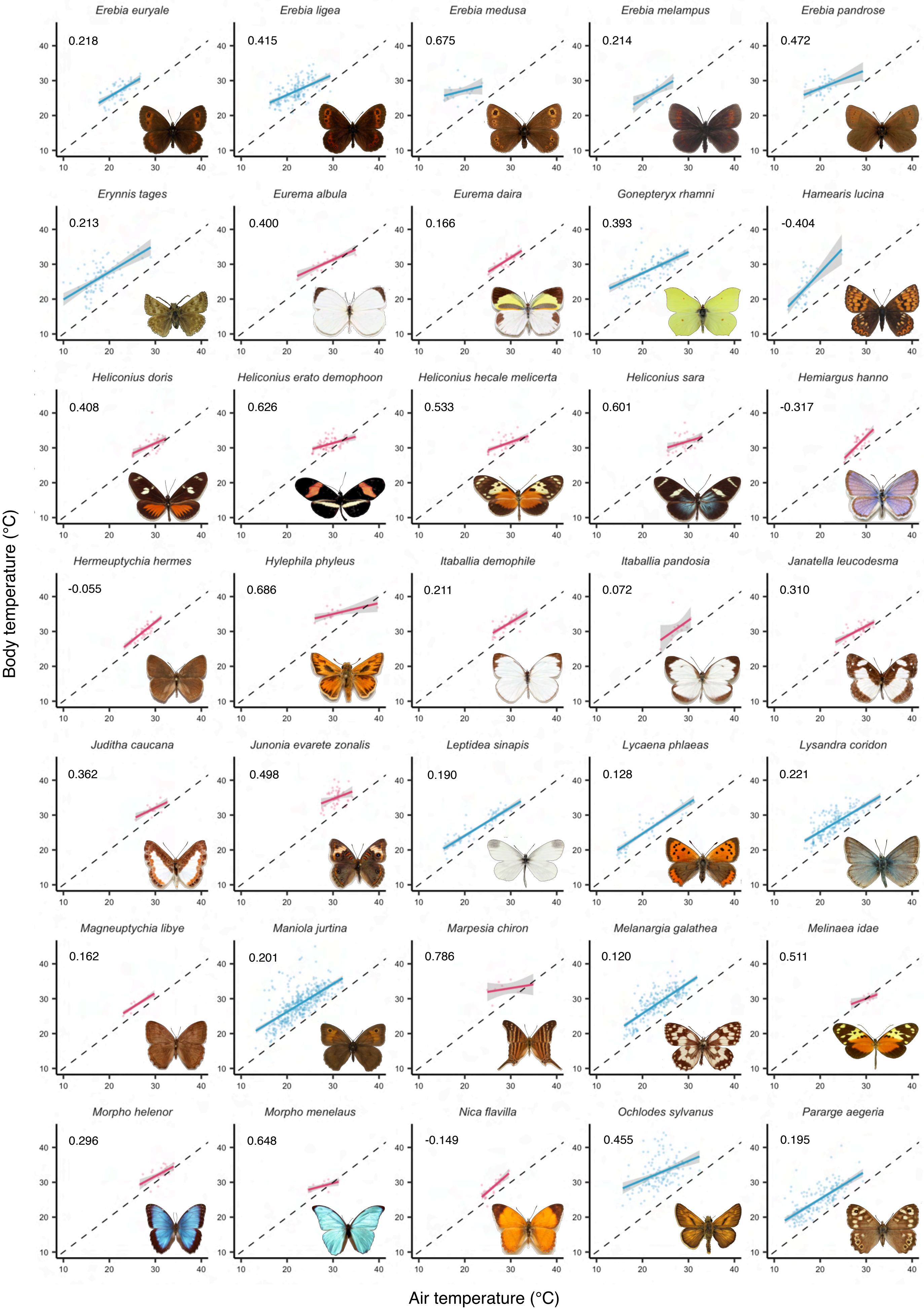
Erebia euryale
0.218
Erebia ligea
0.415
Erebia medusa
0.675
Erebia melampus
0.214
Erebia pandrose
0.472
Erynnis tages
0.213
Eurema albula
0.400
Eurema daira
0.166
Gonepteryx rhamni
0.393
Hamearis lucina
-0.404
Heliconius doris
0.408
Heliconius erato demophoon
0.626
Heliconius hecale melicerta
0.533
Heliconius sara
0.601
Hemiargus hanno
-0.317
Hermeuptychia hermes
-0.055
Hylephila phyleus
0.686
Itaballia demophile
0.211
Itaballia pandosia
0.072
Janatella leucodesma
0.310
Juditha caucana
0.362
Junonia evarete zonalis
0.498
Leptidea sinapis
0.190
Lycaena phlaeas
0.128
Lysandra coridon
0.221
Magneuptychia libye
0.162
Maniola jurtina
0.201
Marpesia chiron
0.786
Melanargia galathea
0.120
Melinaea idae
0.511
Morpho helenor
0.296
Morpho menelaus
0.648
Nica flavilla
-0.149
Ochlodes sylvanus
0.455
Pararge aegeria
0.195
Body temperature (°C)
Air temperature (°C)
10
20
30
40

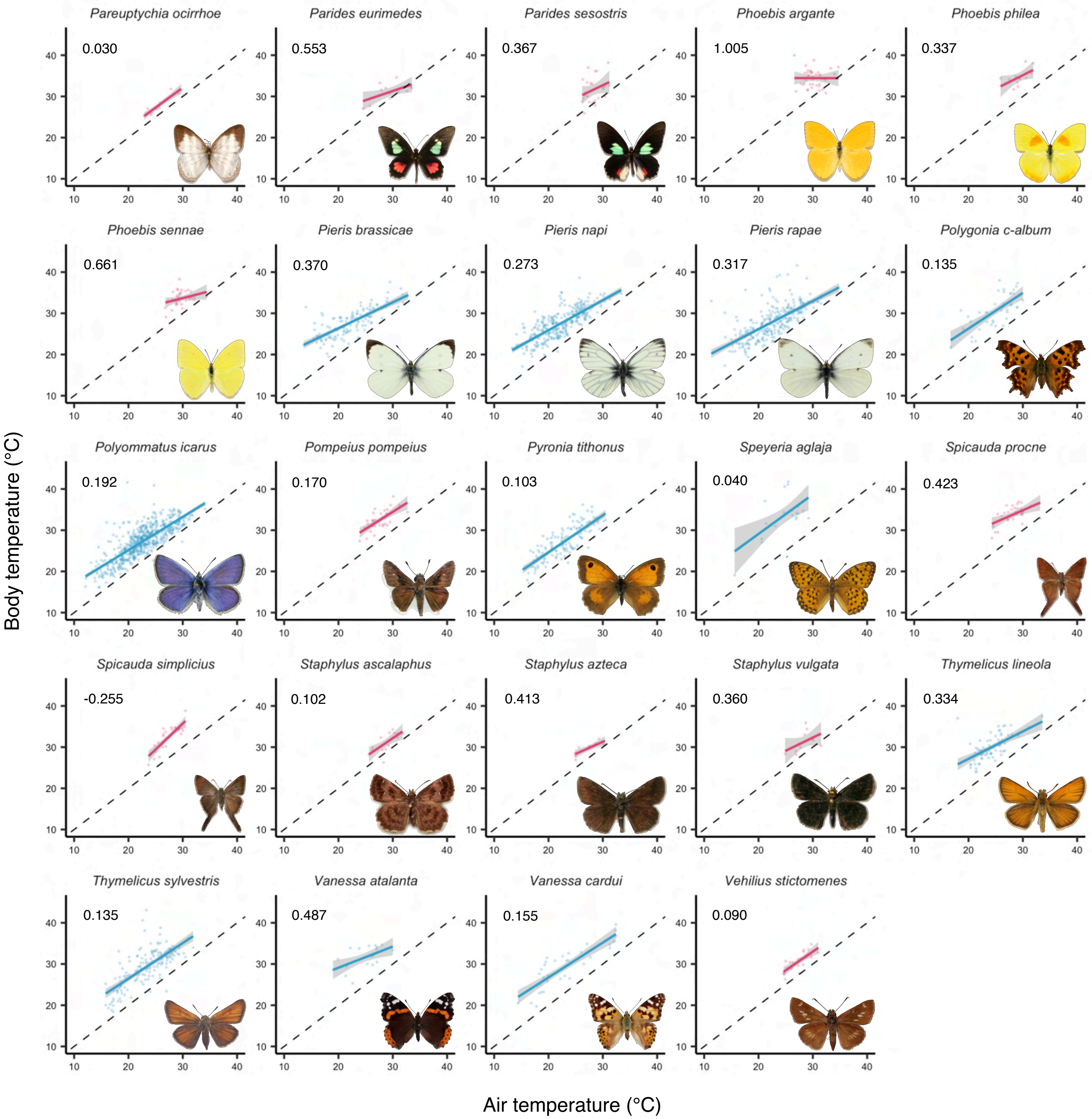

SUPPLEMENTARY FIGURE 2. *Individual butterfly body temperatures (°C) at different air temperatures (°C) from neotropical (pink) and temperate (blue) regions for each species. Solid lines represent the modelled relationship between body temperature and air temperature. The grey bands show the 95% confidence intervals. The black dashed line represents a 1:1 relationship between body and air temperature. The buffering ability estimate is in the top left corner and is calculated as the slope of a simple linear regression, with body temperature as the response variable and air temperature as the predictor variable, subtracted from one. See Supplementary Table 5 for inset photo credits. Photos are not to scale.*

SUPPLEMENTARY TABLE 5. *Photo credits for inset photos in Supplementary Fig. 2. Where required permission of use has been obtained from the author*

Species	Photo credit	License
TEMPERATE		
Aglais io	Malinen, Pekka, Luomus	CC BY-SA 4.0
Aglais urticae	Malinen, Pekka, Luomus	CC BY-SA 4.0
Anthocharis cardamines	Pekka Malinen	CC BY-SA 4.0
Aphantopus hyperantus	Malinen, Pekka, Luomus	CC BY-SA 4.0
Araschnia levana	Eino Ylönen	CC BY-SA 4.0
Argynnis paphia	Eino Ylönen	CC BY-SA 4.0
Aricia agestis	Bartoňová, Beneš, Faltýnek, Konvička. https://doi.org/10.3897/nl.42.38853.figure2	CC BY 4.0
Carterocephalus palaemon	Malinen, Pekka, Luomus	CC BY-SA 4.0
Celastrina argiolus	Jani Järvi, Jani Järvi	CC BY-SA 4.0
Coenonympha pamphilus	Pekka Malinen	CC BY-SA 4.0
Colias hyale	Malinen, Pekka, Luomus	CC BY-SA 4.0
Cupido minimus	Malinen, Pekka, Luomus	CC BY-SA 4.0
Erebia aethiops	M. Fatahi	All rights reserved - please ask for the terms of use if required
Erebia epiphron	M. Fatahi	All rights reserved - please ask for the terms of use if required
Erebia euryale	Malinen, Pekka, Luomus	CC BY-SA 4.0
Erebia ligea	Malinen, Pekka, Luomus	CC BY-SA 4.0
Erebia medusa	Malinen, Pekka, Luomus	CC BY-SA 4.0
Erebia melampus	Paolo Palmi	All rights reserved - please ask for the terms of use if required
Erebia pandrose	Malinen, Pekka, Luomus	CC BY-SA 4.0
Erynnis tages	Natural History Museum (2014). Specimens (from Collection specimens) [BNHH (E) 1338624]	© Images copyright Trustees Natural History Museum
Gonepteryx rhamni	Malinen, Pekka, Luomus	CC BY-SA 4.0
Hamearis lucina	Paolo Palmi	All rights reserved - please ask for the terms of use if required
Leptidea sinapis	Malinen, Pekka, Luomus	CC BY-SA 4.0
Lycaena phlaeas	Malinen, Pekka, Luomus	CC BY-SA 4.0
Lysandra coridon	Paolo Palmi	All rights reserved - please ask for the terms of use if required
Maniola jurtina	Pekka Malinen	CC BY-SA 4.0
Melanargia galathea	M. Fatahi	All rights reserved - please ask for the terms of use if required
Ochlodes sylvanus	Malinen, Pekka, Luomus	CC BY-SA 4.0
Pararge aegeria	Malinen, Pekka, Luomus	CC BY-SA 4.0
Pieris brassicae	Malinen, Pekka, Luomus	CC BY-SA 4.0
Pieris napi	Malinen, Pekka, Luomus	CC BY-SA 4.0
Pieris rapae	Malinen, Pekka, Luomus	CC BY-SA 4.0
Polygonia c-album	Malinen, Pekka, Luomus	CC BY-SA 4.0
Polyommatus icarus	Pekka Malinen	CC BY-SA 4.0
Pyronia tithonus	Katharina Schneeberg	All rights reserved - please ask for the terms of use if required. Specimen from the collection of POLLICHIA
Speyeria aglaja	Eino Ylönen	CC BY-SA 4.0
Thymelicus lineola	Pekka Malinen	CC BY-SA 4.0
Thymelicus sylvestris	Paolo Palmi	All rights reserved - please ask for the terms of use if required
Vanessa atalanta	J. Tyllinen	CC BY-SA 4.0
Vanessa cardui	Malinen, Pekka, Luomus	CC BY-SA 4.0
NEOTROPICAL		
Adelpha cytherea	Kim Davis, Mike Strangeland, Andrew Warren 2009	CC BY-SA 4.0
Aides dysoni	Bernard Hermier	© Images copyright Trustees Natural History Museum
Anartia fatima	D.H. Janzen, W. Hallwachs 2011	CC BY-SA 4.0
Anartia jatrophae	Kim Davis, Mike Strangeland, Andrew Warren 2009	CC BY-SA 4.0
Anthanassa tulcis	Jim P. Brock 2009	CC BY-SA 4.0
Aphrissa statira	Andrew D. Warren 2011	CC BY-SA 4.0
Arawacus togarna	Kim Davis, Mike Strangeland, Andrew Warren 2008	CC BY-SA 4.0
Battus polydamas	Kim Davis, Mike Strangeland 2005	CC BY-SA 4.0
Burnsius orcus	Kim Davis, Mike Strangeland, Andrew Warren 2009	CC BY-SA 4.0
Calephelis sp	Kim Davis, Mike Strangeland, Andrew Warren 2008	CC BY-SA 4.0
Cecropterus dorantes	Kim Davis, Mike Strangeland, Andrew Warren 2009	CC BY-SA 4.0
Chlosyne lacinia	Kim Davis, Mike Strangeland 2006	CC BY-SA 4.0
Cissia pompilia	Kim Davis, Mike Strangeland 2006	CC BY-SA 4.0
Cissia terrestris	Nick V. Grishin 2010	CC BY-SA 4.0
Cupido comyntas	Kim Davis, Mike Strangeland, Andrew Warren 2010	CC BY-SA 4.0
Danaus plexippus	Andrew Warren 2011	CC BY-SA 4.0
Detritivora hermodora	Kim Davis, Mike Strangeland, Andrew Warren 2008	CC BY-SA 4.0

Dione juno	Kim Davis, Mike Strangeland 2005	CC BY-SA 4.0
Dione moneta	Andrew Warren 2010	CC BY-SA 4.0
Dryas iulia	Kim Davis, Mike Strangeland, Andrew Warren 2009	CC BY-SA 4.0
Dynamine paulina	Kim Davis, Mike Strangeland, Andrew Warren 2009	CC BY-SA 4.0
Eurema albula	Andrew D. Warren 2011	CC BY-SA 4.0
Eurema daira	Kim Davis, Mike Strangeland, Andrew Warren 2009	CC BY-SA 4.0
Heliconius doris	Kim Davis, Mike Strangeland, Andrew Warren 2010	CC BY-SA 4.0
Heliconius erato	Gerardo Lamas	© Images copyright Trustees Natural History Museum
Heliconius hecale	Kim Davis, Mike Strangeland, Andrew Warren 2009	CC BY-SA 4.0
Heliconius sara	Kim Davis, Mike Strangeland, Andrew Warren 2009	CC BY-SA 4.0
Hemiargus hanno	Kim Davis, Mike Strangeland, Andrew Warren 2009	CC BY-SA 4.0
Hermeuptychia hermes	Kim Davis, Mike Strangeland 2006	CC BY-SA 4.0
Hylephila phyleus	Jim P. Brock 2009	CC BY-SA 4.0
Itaballia demophile	Kim Davis, Mike Strangeland, Andrew Warren 2010	CC BY-SA 4.0
Itaballia pandosia	Kim Davis, Mike Strangeland, Andrew Warren 2009	CC BY-SA 4.0
Janatella leucodesma	Kim Davis, Mike Strangeland, Andrew Warren 2009	CC BY-SA 4.0
Juditha caucana	Kim Davis, Mike Strangeland, Andrew Warren 2010	CC BY-SA 4.0
Junonia zonalis	John Calhoun 2010	CC BY-SA 4.0
Magneuptychia libye	Jim P. Brock 2011	CC BY-SA 4.0
Marpesia chiron	Andrew Warren 2011	CC BY-SA 4.0
Melinaea idae	Keith Willmott 2010	CC BY-SA 4.0
Morpho helenor	Kim Davis, Mike Strangeland, Andrew Warren 2009	CC BY-SA 4.0
Morpho menelaus	Kim Davis, Mike Strangeland, Andrew Warren 2009	CC BY-SA 4.0
Nica flavilla	Kim Davis, Mike Strangeland, Andrew Warren 2009	CC BY-SA 4.0
Pareuptychia ocirrhoe	Kim Davis, Mike Strangeland 2010	CC BY-SA 4.0
Parides eurimedes	Jim P. Brock 2009	CC BY-SA 4.0
Parides sesostris	Kim Davis, Mike Strangeland, Andrew Warren 2008	CC BY-SA 4.0
Phoebis argante	Andrew D. Warren 2011	CC BY-SA 4.0
Phoebis philea	Kim Davis, Mike Strangeland, Andrew Warren 2009	CC BY-SA 4.0
Phoebis sennae	Andrew D. Warren 2011	CC BY-SA 4.0
Pompeius pompeius	Andrew D. Warren 2007	CC BY-SA 4.0
Spicauda procne	Kim Davis, Mike Strangeland, Andrew Warren 2009	CC BY-SA 4.0
Spicauda simplicius	Nick V. Grishin 2013	CC BY-SA 4.0
Staphylus ascalaphus	Kim Davis, Mike Strangeland, Andrew Warren 2009	CC BY-SA 4.0
Staphylus azteca	D.H. Janzen, W. Hallwachs 2010	CC BY-SA 4.0
Staphylus vulgata	D.H. Janzen, W. Hallwachs 2010	CC BY-SA 4.0
Vehilius stictomenes	Bernard Hermier	CC BY-SA 4.0

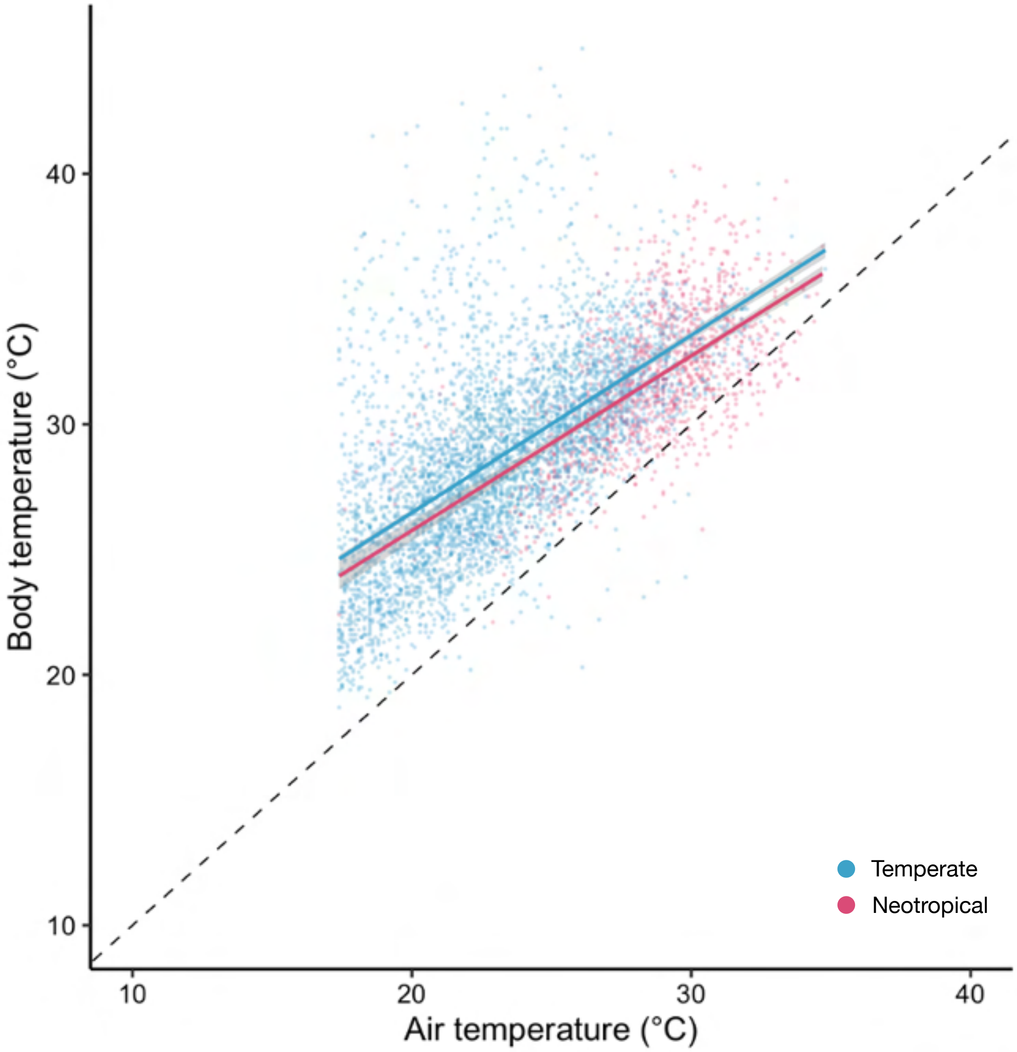

SUPPLEMENTARY FIGURE 3. *Individual butterfly body temperatures (°C) at different air temperatures (°C) from neotropical (pink) and temperate (blue) regions, restricted to only include air temperatures within the range experienced in both regions. Solid lines represent the modelled relationship between body temperature and air temperature. The grey bands show the 95% confidence intervals. The black dashed line represents a 1:1 relationship between body and air temperature.*

SUPPLEMENTARY RESULTS 2. Buffering ability - controlling for differences in air temperature between neotropical and temperate regions across the full assemblage.

When the data were restricted to only include T_a within the range experienced in both regions, the mean buffering estimate was higher for neotropical butterflies than temperate butterflies (neotropical butterflies: 0.331 ± 0.034; temperate butterflies 0.249 ± 0.011; $\chi^2 = 5.673$, df = 1, $p = 0.017$; Supplementary Fig. 3).

SUPPLEMENTARY TABLE 6. *Buffering ability estimates, standard error (SE) and number of species (n) per family for temperate and neotropical families. Results of the likelihood ratio test between models with and without the two-way interaction between region and air temperature are given (test-statistic (χ^2), degrees of freedom (df) and likelihood p-value). Statistically significant likelihood p values are marked with an asterisk and indicate a significant difference in buffering ability between neotropical and temperate regions for species in that family.*

Family	Temperate			Neotropical			χ^2	df	Likelihood p-value
	Buffering ability estimate	SE	n	Buffering ability estimate	SE	n			
Hesperiidae	0.269	0.039	5	0.212	0.099	11	0.346	1	0.557
Lycaenidae	0.197	0.017	6	-0.020	0.149	3	2.021	1	0.155
Nymphalidae	0.180	0.015	21	0.397	0.047	26	21.663	1	< 0.001*
Pieridae	0.326	0.019	7	0.426	0.092	8	1.101	1	0.294
Riodinidae	-0.404	0.181	1	0.373	0.233	3	11.831	1	< 0.001*

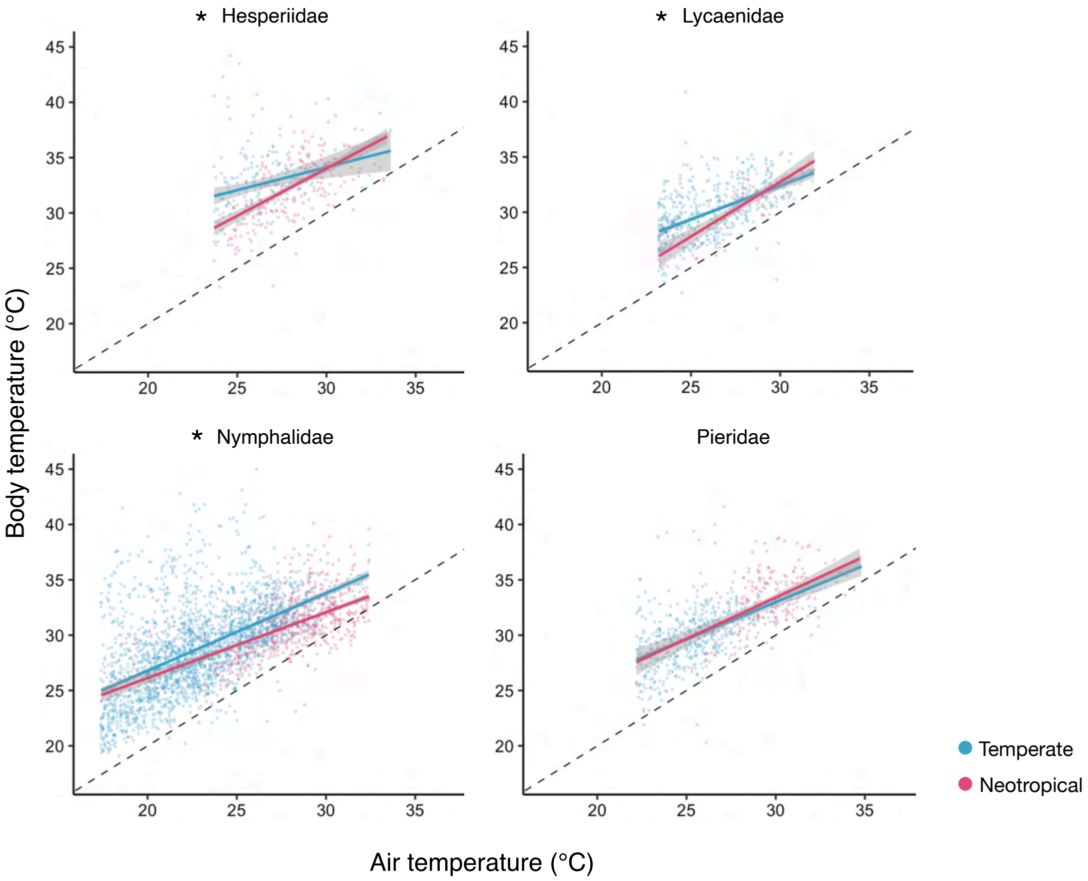

SUPPLEMENTARY FIGURE 4. *Individual butterfly body temperatures (°C) at different air temperatures (°C) from neotropical (pink) and temperate (blue) regions for each taxonomic family, restricted to only include air temperatures within the range experienced in both regions. Solid lines represent the modelled relationship between body temperature and air temperature. The grey bands show the 95% confidence intervals. The black dashed line represents a 1:1 relationship between body and air temperature. Families with a significant difference in buffering ability estimate between neotropical and temperate species are marked with an asterisk.*

SUPPLEMENTARY TABLE 7. *Buffering ability estimates, standard error (SE) and number of species (n) per family for temperate and tropical families. To control for differences in air temperature between neotropical and temperate regions, data were restricted to the air temperature range experienced in both tropical and temperate regions. Results of the likelihood ratio test between models with and without the two-way interaction between region and air temperature are given (test-statistic (χ^2), degrees of freedom (df) and likelihood p-value). Statistically significant p values are marked with an asterisk and indicate a significant difference in buffering ability between neotropical and temperate regions for species in that family.*

Family	Temperate			Neotropical			χ^2	df	Likelihood p-value
	Buffering ability estimate	SE	n	Buffering ability estimate	SE	n			
Hesperiidae	0.588	0.092	3	0.134	0.122	11	13.765	1	< 0.001*
Lycaenidae	0.390	0.038	6	0.011	0.129	3	8.594	1	0.003*
Nymphalidae	0.221	0.017	21	0.361	0.052	23	7.393	1	0.007*
Pieridae	0.301	0.043	7	0.415	0.091	8	1.449	1	0.229

SUPPLEMENTARY RESULTS 3. Buffering ability - controlling for differences in air temperature between neotropical and temperate regions by family.

Temperate Hesperiidae and temperate Lycaenidae were better at buffering their T_b against changes in T_a than Hesperiidae and Lycaenidae from neotropical regions (Hesperiidae: $\chi^2 = 13.765$, df = 1, $p < 0.001$; Lycaenidae: $\chi^2 = 8.594$, df = 1, $p = 0.003$) (Supplementary Fig. 4, Supplementary Table 7). Neotropical Nymphalidae were better at buffering their T_b against changes in T_a than Nymphalidae from temperate regions ($\chi^2 = 7.393$, df = 1, $p = 0.007$) (Supplementary Fig. 4, Supplementary Table 7). There was no difference in the buffering ability of Pieridae from neotropical and temperate regions ($\chi^2 = 1.445$, df = 1, $p = 0.229$). There were insufficient data on Riodinidae species to undertake this analysis.

SUPPLEMENTARY TABLE 8: *Output from the best fitting linear mixed effects model, with body temperature (T_b) as the predictor variable and air temperature (T_a), region (neotropical and temperate), mean forewing length, mean forewing aspect ratio and the two-way interactions between Ta and each of mean forewing length and mean forewing aspect ratio as response variables. Data were restricted to the air temperature range experienced in both tropical and temperate regions.Mean forewing length was log_{10} transformed to ensure it was on the same scale as other predictor variables. Species was included as a random effect. Significant terms are marked with an asterix*

	Estimate	SE	df	F	*p*
Region: temperate	-11.643	4.947	1	18.390	<0.001*
Region: tropical	-10.264	0.322			
T_a	1.491	0.168	1	78.403	<0.001*
Mean forewing length	3.140	1.012	1	9.623	0.002*
Mean forewing aspect ratio	8.129	2.452	1	10.991	<0.001*
T_a : Mean forewing length	-0.212	0.079	1	7.258	0.007*
T_a : Mean forewing aspect ratio	-0.274	0.083	1	11.000	<0.001*

SUPPLEMENTARY TABLE 9. *Mean values for microclimate selection, postural thermoregulation and index of thermal specialisation, the corresponding standard errors (SE) and number of species (n) across the whole assemblage and per family for temperate and neotropical regions. Results of the likelihood ratio test between models with and without the two-way region:air* temperature interaction, and the region term, are given (test-statistic (χ2), degrees of freedom (df) and likelihood p-value). Statistically significant p-values are marked with an asterisk and *indicate a significant difference in the use of microclimate selection or postural thermoregulation, or in the index of thermal specialisation, between neotropical and temperate regions for species across the assemblage or within one family. In these cases, the higher value is marked in bold.*

Microclimate selection

Group	Temperate			Neotropical			Region:air temperature interaction			Region + air temperature		
	x̄	SE	n	x̄	SE	n	χ^2	df	Likelihood p-value	χ^2	df	Likelihood p-value
Assemblage	1.298	0.070	26	0.846	0.135	13	0.491	1	0.484	0.104	1	0.747
Hesperiidae	1.329	0.234	3	0.674	0.229	5	1.890	1	0.169	0.025	1	0.875
Nymphalidae	1.400	0.105	15	0.879	0.178	7	0.283	1	0.595	0.007	1	0.932

Postural thermoregulation

Group	Temperate			Neotropical			Region:air temperature interaction			Region + air temperature		
	x̄	SE	n	x̄	SE	n	χ^2	df	Likelihood p-value	χ^2	df	Likelihood p-value
Assemblage	**4.220**	0.109	26	2.200	0.171	13	0.075	1	0.785	8.314	1	0.004*
Hesperiidae	6.259	0.525	3	2.726	0.357	5	0.239	1	0.625	2.431	1	0.119
Nymphalidae	**4.723**	0.151	15	2.103	0.200	7	0.601	1	0.438	11.855	1	<0.001*

Index of thermal specialisation

Group	Temperate			Neotropical			Region:air temperature interaction			Region + air temperature		
	x̄	SE	n	x̄	SE	n	χ^2	df	Likelihood p-value	χ^2	df	Likelihood p-value
Assemblage	**2.922**	0.144	26	1.355	0.269	13	0.000	1	0.997	7.15	1	0.008*
Hesperiidae	4.930	0.531	3	2.053	0.523	5	0.059	1	0.809	2.516	1	0.113
Nymphalidae	**3.323**	0.210	15	1.224	0.328	7	0.037	1	0.848	7.97	1	0.005*

SUPPLEMENTARY RESULTS 4. Microclimate selection, postural thermoregulation and ITS - controlling for differences in air temperature between neotropical and temperate regions.

When data were restricted to the range of air temperatures which occurred in both regions, microclimate selection, postural thermoregulation and the ITS did not significantly differ between neotropical and temperate regions (microclimate selection: χ^2 = 0.001, df = 1, p = 0.972; postural thermoregulation: χ^2 = 3.142, df = 1, p = 0.076; ITS: χ^2 = 2.053, df = 1, p = 0.152, Supplementary Fig. 5, Supplementary Table 10). This was the same for Nymphalidae when tested separately for microclimate selection (χ^2 = 0.085, df = 1, p = 0.771) and ITS (χ^2 = 3.147, df = 1, p = 0.076), but temperate Nymphalidae butterflies used postural thermoregulation to increase their temperature significantly more than neotropical butterflies (χ^2 = 6.445, df = 1, p = 0.011, Supplementary Table 10). There was insufficient data to undertake this analysis on the other families.

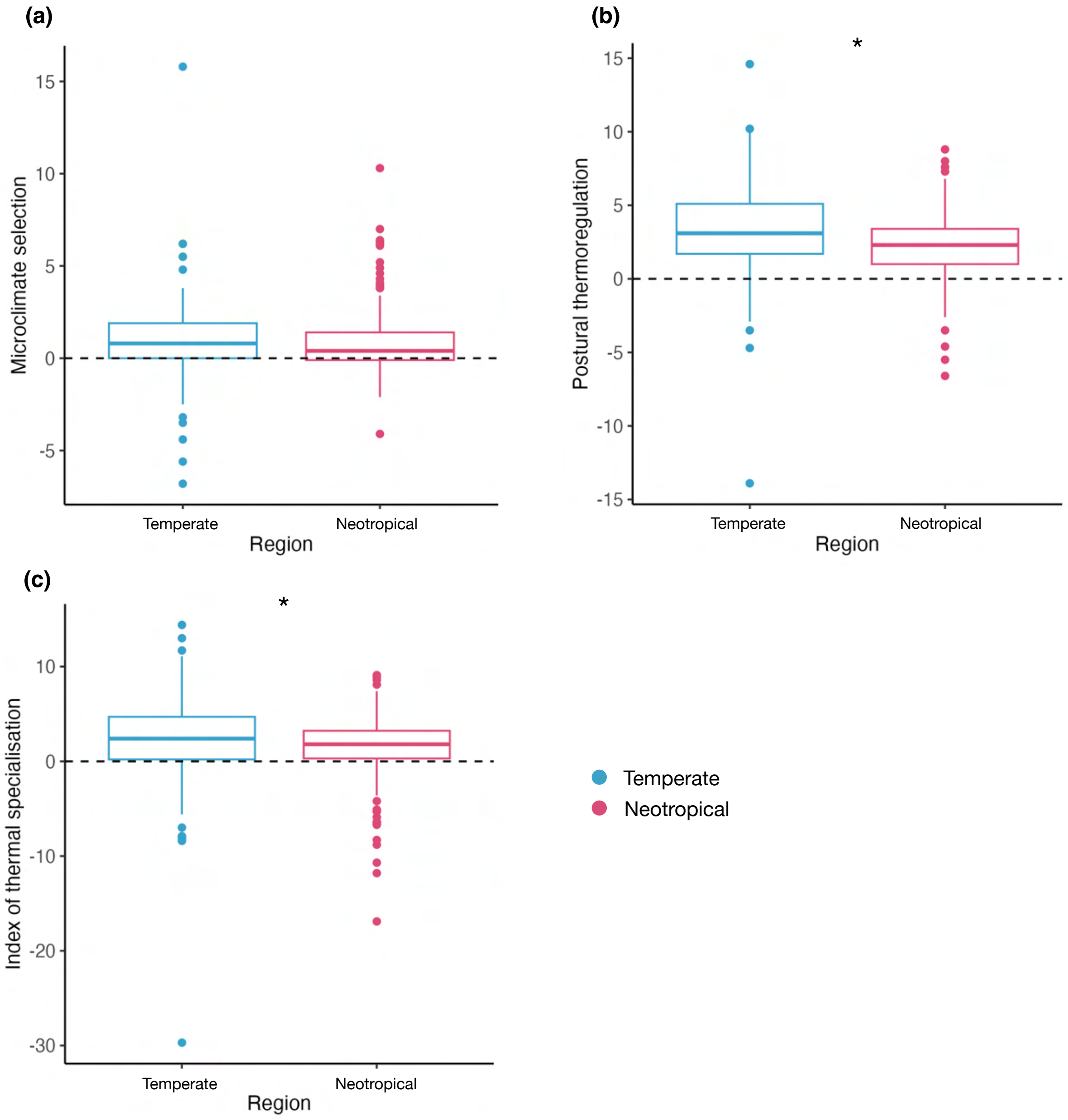

SUPPLEMENTARY FIGURE 5: *Microclimate selection (the difference between microclimate temperature and air temperature, a), postural thermoregulation (the difference between body temperature and microclimate temperature, b) and index of thermal specialisation (postural thermoregulation – microclimate selection, c) of temperate (blue) and neotropical (pink) butterflies. Data restricted to only include air temperatures within the range experienced in both regions. Significant differences between neotropical and temperate butterflies are marked with an asterisk.*

SUPPLEMENTARY TABLE 10. *Mean values for microclimate selection, postural thermoregulation and index of thermal specialisation, the corresponding standard errors (SE) and number of species (n) across the whole assemblage and per family for temperate and neotropical regions. Results of the likelihood ratio test between models with and without the two-way region:air temperature interaction, and the region term, are given (test-statistic (χ^2), degrees of freedom (df) and likelihood p-value). Statistically significant p-values are marked with an asterisk and indicate a significant difference in the use of microclimate selection or postural thermoregulation, or in the index of thermal specialisation, between neotropical and temperate regions for species across the assemblage or within one family. In these cases, the higher value is marked in bold. To control for differences in air temperature between neotropical and temperate regions, data were restricted to the air temperature range experienced in both neotropical and temperate regions.*

Microclimate selection

Group	Temperate			Neotropical			Region:air temperature interaction			Region + air temperature		
	x̄	SE	n	x̄	SE	n	χ^2	df	Likelihood p-value	χ^2	df	Likelihood p-value
Assemblage	0.943	0.122	14	0.846	0.135	13	1.292	1	0.256	0.001	1	0.972
Nymphalidae	0.969	0.223	7	0.879	0.178	7	0.103	1	0.749	0.085	1	0.771

Postural thermoregulation

Group	Temperate			Neotropical			Region:air temperature interaction			Region + air temperature		
	x̄	SE	n	x̄	SE	n	χ^2	df	Likelihood p-value	χ^2	df	Likelihood p-value
Assemblage	3.216	0.179	14	2.200	0.171	13	1.223	1	0.269	3.142	1	0.076
Nymphalidae	**3.845**	0.310	7	2.103	0.200	7	3.243	1	0.072	6.445	1	0.011*

Index of thermal specialisation

Group	Temperate			Neotropical			Region:air temperature interaction			Region + air temperature		
	x̄	SE	n	x̄	SE	n	χ^2	df	Likelihood p-value	χ^2	df	Likelihood p-value
Assemblage	2.273	0.264	14	1.355	0.269	13	0.090	1	0.764	2.053	1	0.152
Nymphalidae	2.876	0.488	7	1.224	0.327	7	0.757	1	0.384	3.147	1	0.076
